# Combining NGN2 programming and dopaminergic patterning for a rapid and efficient generation of hiPSC-derived midbrain neurons

**DOI:** 10.1038/s41598-022-22158-4

**Published:** 2022-10-13

**Authors:** Razan Sheta, Maxime Teixeira, Walid Idi, Marion Pierre, Aurelie de Rus Jacquet, Vincent Emond, Cornelia E. Zorca, Benoît Vanderperre, Thomas M. Durcan, Edward A. Fon, Frédéric Calon, Mohamed Chahine, Abid Oueslati

**Affiliations:** 1 CHU de Québec Research Center, Axe Neurosciences, Quebec City, Canada; 2 Department of Molecular Medicine, Faculty of Medicine, Université Laval, Quebec City, Canada; 3 CERVO Brain Research Center, 2601, rue de La Canardière, Quebec City, Canada; 4 Department of Psychiatry and Neurosciences, Faculty of Medicine, Université Laval, Quebec City, Canada; 5 McGill Parkinson Program and Neurodegenerative Diseases Group, Montreal Neurological Institute, McGill University, Montreal, Canada; 6 The Neuro’s Early Drug Discovery Unit (EDDU), Montreal Neurological Institute, McGill University, Montreal, Canada; 7 Département des sciences biologiques, Université du Québec à Montréal, Montreal, QC Canada; 8 Centre d’Excellence en Recherche sur les Maladies Orphelines – Fondation Courtois (CERMO-FC), Montreal, Canada; 9 Faculty of Pharmacy, Université Laval, Quebec City, Canada; 10 Department of Medicine, Faculty of Medicine, Université Laval, Quebec City, Canada

**Keywords:** Cellular neuroscience, Diseases of the nervous system, Stem cells in the nervous system, Parkinson's disease

## Abstract

The use of human derived induced pluripotent stem cells (hiPSCs) differentiated to dopaminergic (DA) neurons offers a valuable experimental model to decorticate the cellular and molecular mechanisms of Parkinson’s disease (PD) pathogenesis. However, the existing approaches present with several limitations, notably the lengthy time course of the protocols and the high variability in the yield of DA neurons. Here we report on the development of an improved approach that combines neurogenin-2 programming with the use of commercially available midbrain differentiation kits for a rapid, efficient, and reproducible directed differentiation of hiPSCs to mature and functional induced DA (iDA) neurons, with minimum contamination by other brain cell types. Gene expression analysis, associated with functional characterization examining neurotransmitter release and electrical recordings, support the functional identity of the iDA neurons to A9 midbrain neurons. iDA neurons showed selective vulnerability when exposed to 6-hydroxydopamine, thus providing a viable in vitro approach for modeling PD and for the screening of small molecules with neuroprotective proprieties.

## Introduction

Parkinson's disease (PD), the most common movement disorder, is characterized by the massive and progressive loss of dopaminergic (DA) neurons from the substantia nigra *pars compacta* (SN*pc*)^1^. The exact mechanisms behind PD pathogenesis and the vulnerability of the DA neuronal population are not fully elucidated^2,3^. In the last decade, the application of strategies for the differentiation of human induced pluripotent stem cells (hiPSCs) into DA neurons have shown great promise for providing a suitable in vitro approach for PD modeling and drug screening^4^.

Many of these protocols are based on the use of chemical compounds, transcription factors programming, and signaling pathway activation^5–10^. One of the most efficient and highly exploited protocol for the generation of DA neurons applies a combination of pharmacological compounds, as well as recombinant proteins^8^. This protocol, by Kirks et al., has been widely used as the basis of many studies optimizing strategies for the generation of DA neurons^11–14^. However, these strategies present with two major limitations. First, they require long protocols for the generation of functional and mature DA neurons (between day in vitro (DIV) 40 and DIV 90). Second, the low purity of the DA culture, with varying amounts of DA neurons as percentage fluctuates between 15 and 40% at early time points (DIV 20 and DIV 30), and 50% to 80% between DIV 70 and DIV 90^8,11–15^.

Recently, the description of a new approach of induced neuronal cell differentiation through the programming by the transcription factor neurogenin-2 (*NGN2)* has shown to efficiently induce rapid differentiation of multipopulational neurons, referred to as iNeurons^16^. This approach has opened doors for regional and specific neuronal differentiation; and several groups have made use of this application to generate cultures with high purity excitatory neurons^16–23^.

Inspired by this work, here we combined *NGN2* programing and the use of commercially available midbrain differentiation kits to force iNeurons differentiation efficiently and rapidly into mature and functional DA (iDA) neurons. We used immunocytochemistry (ICC), gene expression analysis, functional characterization examining DA release and electrophysiological recordings to confirm the identity and the functional characteristics of the iDA neurons. Finally, we investigated the utility of our approach to model PD by assessing iDA selective vulnerability to the neurotoxin 6-hydroxydopamine (6-OHDA) and for the screening of small molecules with reported neuroprotective prperties.

## Results

### Combination of *NGN2* overexpression and midbrain differentiation media rapidly induces a dopaminergic phenotype

**Figure 1:**
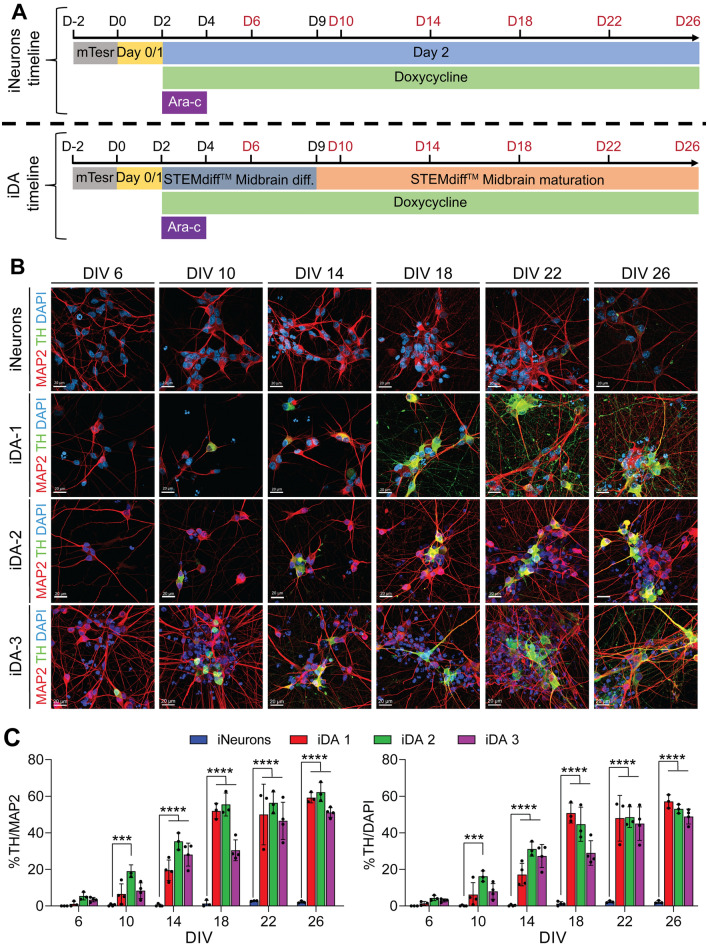
Combination of *NGN2* overexpression and midbrain differentiation and maturation medias force the dopaminergic phenotype of the iPSC-derived iNeurons. (**A**) Schematic timeline of the use of different conditioning media to pattern, differentiate and mature hiPSCs to generate dopaminergic neurons (iDA). The iNeurons are patterned from hiPSCs from DIV 0 to DIV 2 using Day 0/1 media. Starting DIV 2, the cells are exposed to the doxycycline to induce NGN2 expression and neuronal differentiation and cultured in Day 2 media. From DIV 2 to DIV 4, cells were exposed to Ara-C to eliminate non-differentiated cells. To induce the dopaminergic phenotype, cells are exposed to the midbrain differentiating media supplemented with SHH from DIV 2 to DIV 9. Differentiated iDA neurons were then exposed to maturation media allowing them to reach a late maturation stage at DIV 26. (**B**) Representative confocal illustrations of ICCs against TH (green) and MAP2 (red) for iNeurons or iDA neurons from 3 iPSC lines (iDA-1, iDA-2 and iDA-3) at different timepoints. Scale bars: 20 µm. (**C**) Quantitative analysis of the percentage of TH^+^ neurons for iNeurons and iDA-1, iDA-2 and iDA-3 neurons at different timepoints. Data represent the average percentage of TH^+^ neurons normalized against MAP2^+^ positive neurons or total number of DAPI nuclei (n = 4). Statistical differences were assessed performing a 2-way ANOVA Šídák's multiple comparisons test comparing iDA neurons to iNeurons at each time point. ***p < 0.001; ****p < 0.0001.

Previous work reported that *NGN2* overexpression, under doxycycline control, efficiently and rapidly induced hiPSCs differentiation and maturation into heterogenous neuronal populations, with a majority of excitatory neurons, referred to as iNeurons^16^. We took advantage of this rapid neuronal differentiation system and sought to constrain the dopaminergic phenotype of the iNeurons by supplementing cells with predefined commercially available midbrain differentiation and maturation medias (Stemcell technologies). We referred to these cells as induced dopaminergic (iDA) neurons. We used two hiPSC lines stably overexpressing TetO-*NGN2*-T2A-PURO (iDA-1 and iDA-2) and one hiPSC line transduced with lentivirus overexpressing TetO-*NGN2*-T2A-PURO (iDA-3)^16^. We generated the iNeurons as previously described by exposing hiPSCs to doxycycline, 2 days after plating (DIV 2)^16^ (Fig. 1A). Then, Cytosine β-D-arabinofuranoside hydrochloride (Ara-C) was added to the media from DIV 2 to DIV 4 to inhibit the proliferation of dividing cells^18,24^. To induce patterning towards midbrain phenotype, we first incubated hiPSCs in midbrain differentiation media from DIV 2 to DIV 8 and then, this media was replaced by midbrain maturation media (from DIV 9 to DIV 26) (Fig. 1A).

To investigate the efficacy of the midbrain patterning and to determine when cells started to exhibit the dopaminergic phenotype, we analyzed the differentiated hiPSCs by ICC for pan-neuronal markers, microtubule-associated protein 2 (MAP2) and β-III-tubulin, and for the dopaminergic neuronal marker tyrosine hydroxylase (TH), every 4 days post-doxycycline treatment (DIV 6, 10, 14, 18, 22 and 26) (Fig. 1A). Upon differentiation, iNeurons and iDA neurons, from the three hiPSC lines, showed positive staining for MAP2 and β-III-tubulin, as early as DIV 6, thus confirming the rapid hiPSCs differentiation into neurons after doxycycline treatment (Fig. 1B). Interestingly, TH staining revealed that iDA-1, -2 and -3 neurons exhibited TH^+^ signal as early as DIV 6 (~ 1–2% of the total number of cells (DAPI^+^) and (~ 2–3% of neuronal cells (MAP2^+^ and β-III-tubulin^+^) (Fig. 1B,C ;Fig. S1). The number of differentiated hiPSCs with TH^+^ signal significantly increased overtime and doubled every 4 days (5–15% at DIV 10, 20–35% at DIV 14, 40–55% at DIV 18) before reaching a maximum of 60% at DIV 22 and DIV 26 for iDA-1 and iDA-2 and 50% for iDA-3 (Fig. 1C). It is important to mention, that a very small proportion of iNeurons showed TH^+^ staining (< 2% of total cells at DIV 26) (Fig. 1B,C), thus confirming that the use of midbrain differentiation and maturation medias was associated with the induction of DA neurons in the culture. Of note, the use of midbrain differentiation and maturation medias in two neuronal progenitor cells, NPC-1 and NPC-2, did not induce any marked dopaminergic differentiation (< 15%), thus confirming that the combination of *NGN2* overexpression and the midbrain differentiation and maturation medias is required for the rapid and efficient induction of the dopaminergic phenotype (Fig. S2).

Finally, to verify whether the iDA culture could be contaminated by other brain cell types, we assessed the presence of glutamatergic (VGlut1^+^) and serotoninergic (SERT^+^) neurons and we observed a low proportion (15%) of these two cell types in the iDA culture (Fig. S3). Moreover, no glial cells markers (GFAP and Iba1) were detected in the culture (Fig. S3), thus confirming that the protocol generated dopaminergic neurons with a minimum contamination by other brain cell types.

Given the fact that our approach induced a similar rapid and efficient dopaminergic neuronal differentiation in the three hiPSC lines, we used iDA-1 line, which we refereed as iDA neurons, for subsequent analysis.

### iDA neurons present with gene expression patterns reminiscent of midbrain dopaminergic neurons

**Figure 2:**
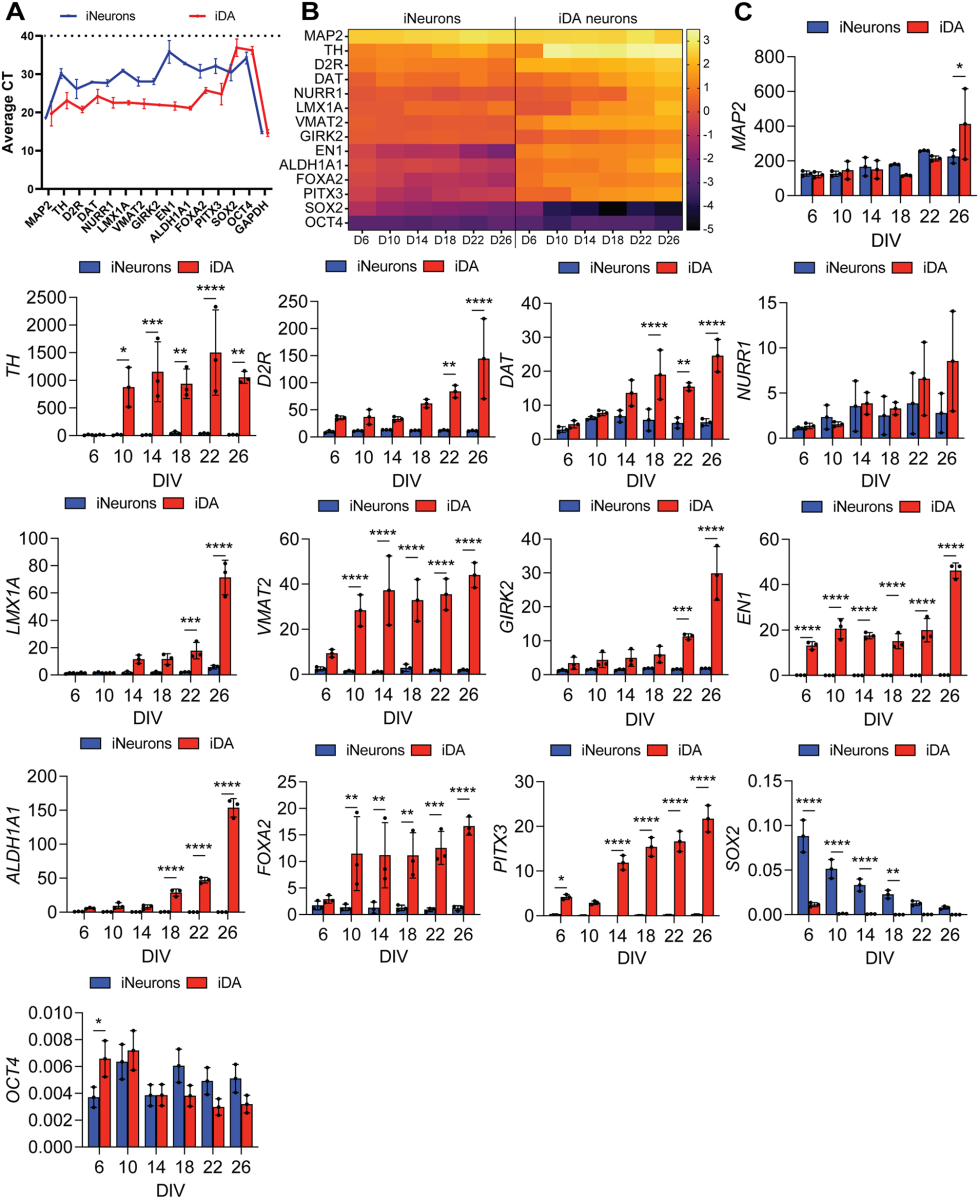
iDA neurons gene expression patterns mimic the gene expression signature of midbrain DA neurons. (**A**) Comparison of gene expression profiles in iNeurons and iDA neurons at DIV 26. The plot represents average CT values of the genes with a cut-off of 40 cycles (n = 3). (**B**) Whole sample quantitative qRT-PCR analysis of the expression levels of the key genes examined. Expression levels, expressed as log changes, are color coded following the color scale on the right. mRNA levels were quantified at each time point for iNeurons or iDA neurons (n = 3). (**C**) Comparison of gene expression profiles in iNeurons and iDA neurons as examined over time, differentiated from iNeurons at DIV 2. The graphs depict the fold-change expression of each gene normalized to the DIV 2 cells (n = 3). Statistical differences were assessed performing a 2-way ANOVA Šídák's multiple comparisons test comparing iDA neurons to iNeurons at each time points. *p < 0.05; **p < 0.01; ***p < 0.001; ****p < 0.0001.

To further characterize the iDA neurons, we performed qRT-PCR analysis to evaluate the genomic expression of additional dopaminergic transcription factors and neuronal markers. Comparison of gene expression profile between iNeurons and iDA, at DIV 26, showed high levels of genes associated with midbrain neuronal differentiation and maturation in iDA neurons^25^ (Fig. 2A). These genes include TH, dopamine D2 receptor (*D2R*), plasma membrane dopamine transporter (*DAT*), nuclear receptor-related 1 protein (*NURR1*), LIM-homeodomain transcription factor (*LMX1A*), vesicular monoamine transporter 2 (*VMAT2*), homeobox protein engrailed-1 (*EN1*), forkhead box protein A2 (*FOXA2*) and midbrain-specific transcription factors pituitary homeobox 3 (*PITX3*) (Fig. 2A). Moreover, qRT-PCR analysis revealed the induction of the gene coding for the G protein-coupled inwardly rectifying potassium channels (*GIRK2*) and aldehyde dehydrogenase (*ALDH1A1*), two commonly used markers for A9 subtype DA neurons, the most vulnerable neuronal population in the midbrain^26,27^ (Fig. 2A). Of note, analysis of the gene expression profile at DIV 26 revealed reproducibility amongst the different iDA lines, in comparison with iNeurons and NPCs, indicating that our protocol allows for a reproducible differentiation of homogenous dopaminergic neuronal population (Fig. S4A,B).

We then assessed gene expression profile overtime, every 4 days starting from DIV 2 in both iNeurons and iDA neurons. As expected, while a very low level of *TH* gene expression was detected in iNeurons, we observed a very early and dramatic increase of *TH* gene expression overtime in iDA neurons (Fig. 2B,C). Moreover, we also observed a significant time-dependent upregulation of key genes associated with midbrain neuronal differentiation and maturation, whereas little or no changes were observed in the iNeurons genes footprint (Fig. 2B,C). Conversely, genes coding for cellular pluripotency such as SRY-Box transcription factor 2 (*SOX2*) and the octamer-binding transcription factor 4 (*Oct4*) were significantly downregulated in both iNeurons and iDA neurons in a time-dependent manner (Fig. 2B,C). Of note, using Western blot analysis, we confirmed that the upregulation of DA-associated genes (Pitx3, LMX-1, EN1 and FOXA2), was correlated with a time-dependent increase of the expression of the corresponding proteins (Fig. S4C). Collectively, these findings confirm the efficient and specific induction of iDA neurons presenting with phenotypic characteristic of mature DA neurons with midbrain identity.

### hiPSC-derived iDA neurons can produce and to release dopamine and metabolites

The rapid and efficient differentiation of hiPSCs into DA neurons prompted us to investigate if these neurons are functional. More specifically, we investigated the capacity of the iDA neurons to produce and to release the neurotransmitter dopamine (DA). Analysis was performed at DIV 18 and DIV 26, the time points when key genes related to dopaminergic transmission were dramatically upregulated (Fig. 2).

**Figure 3:**
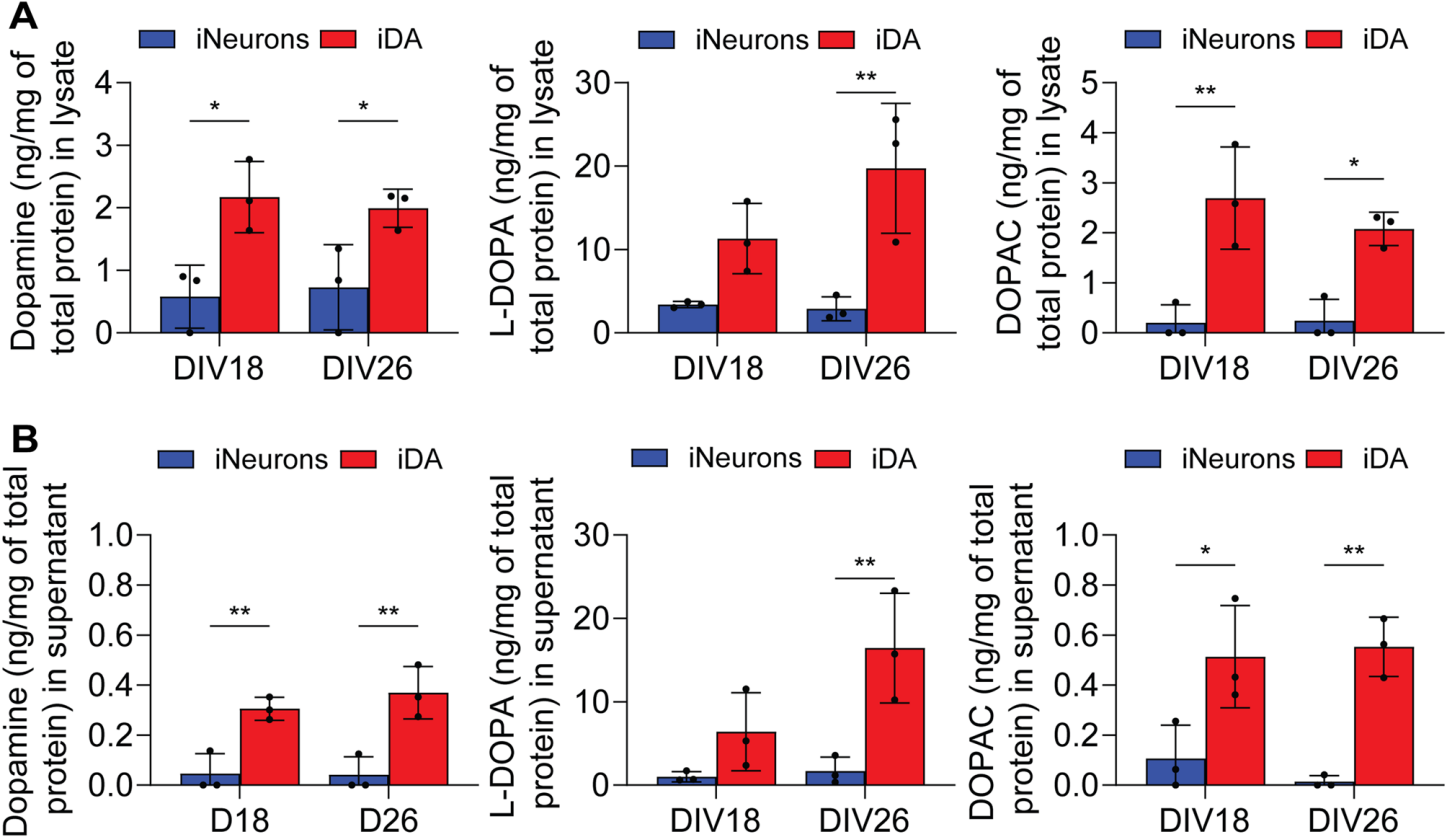
iDA neurons can produce and release dopamine. (**A**) Quantification of the levels of L-DOPA, dopamine and DOPAC in lysates from iNeurons and iDA neurons at DIV 18 and DIV 26 (normalized to total proteins) (n = 3). (**B**) Quantification of the levels of L-DOPA, dopamine and DOPAC in the extracellular culture media of iNeurons and iDA neurons at DIV 18 and DIV 26 (normalized to total proteins) (n = 3). Statistical differences were assessed by performing a 2-way ANOVA followed by a Šídák's multiple comparisons test comparing iDA neurons to iNeurons at each time point. *p < 0.05; **p < 0.01.

First, we investigated the capacity of the iDA neurons to produce DA using high-performance liquid chromatography (HPLC). Analysis included the detection of L-dihydroxyphenylalanine (L-DOPA), DA and dihydroxyphenylacetic acid (DOPAC) levels in the cell lysate. L-DOPA is the TH-produced precursor of DA, while the synthesis of DA itself require the additional activity of L-DOPA decarboxylase. Finally, DOPAC is a metabolite of DA generated by the catalytic action of monoamine oxidase. When compared to iNeurons, we observed significantly higher levels of L-DOPA, DA and DOPAC in the lysate of iDA neurons, suggesting that iDA neurons are indeed capable of dopamine biosynthesis (Fig. 3A).

Then, we investigated the capacity of the iDA neurons to release the DA and metabolites. To this end, we stimulated cells with 56 mM of KCl and analyzed the extracellular media by HPLC. Our data reported the presence of higher concentrations of L-DOPA, DA, and DOPAC in the culture media, compared to iNeurons, thus confirming that iDA neurons can release the endogenously produced neurotransmitter (Fig. 3B). It is important to note that levels of cellular and released DA and metabolite seemed to remain constant between DIV 18 and DIV 26, suggesting that the neurons may have reached a relatively high level of maturity in term of neurotransmitter biosynthesis and release as early as DIV 18 (Fig. 3). Overall, our results support that iDA neurons can rapidly reach maturity by exhibiting key functional features of primary midbrain DA neurons, notably the production and the release of midbrain neurotransmitter, the DA.

### iDA are functional and electrically active 2 weeks post-induction

**Figure 4:**
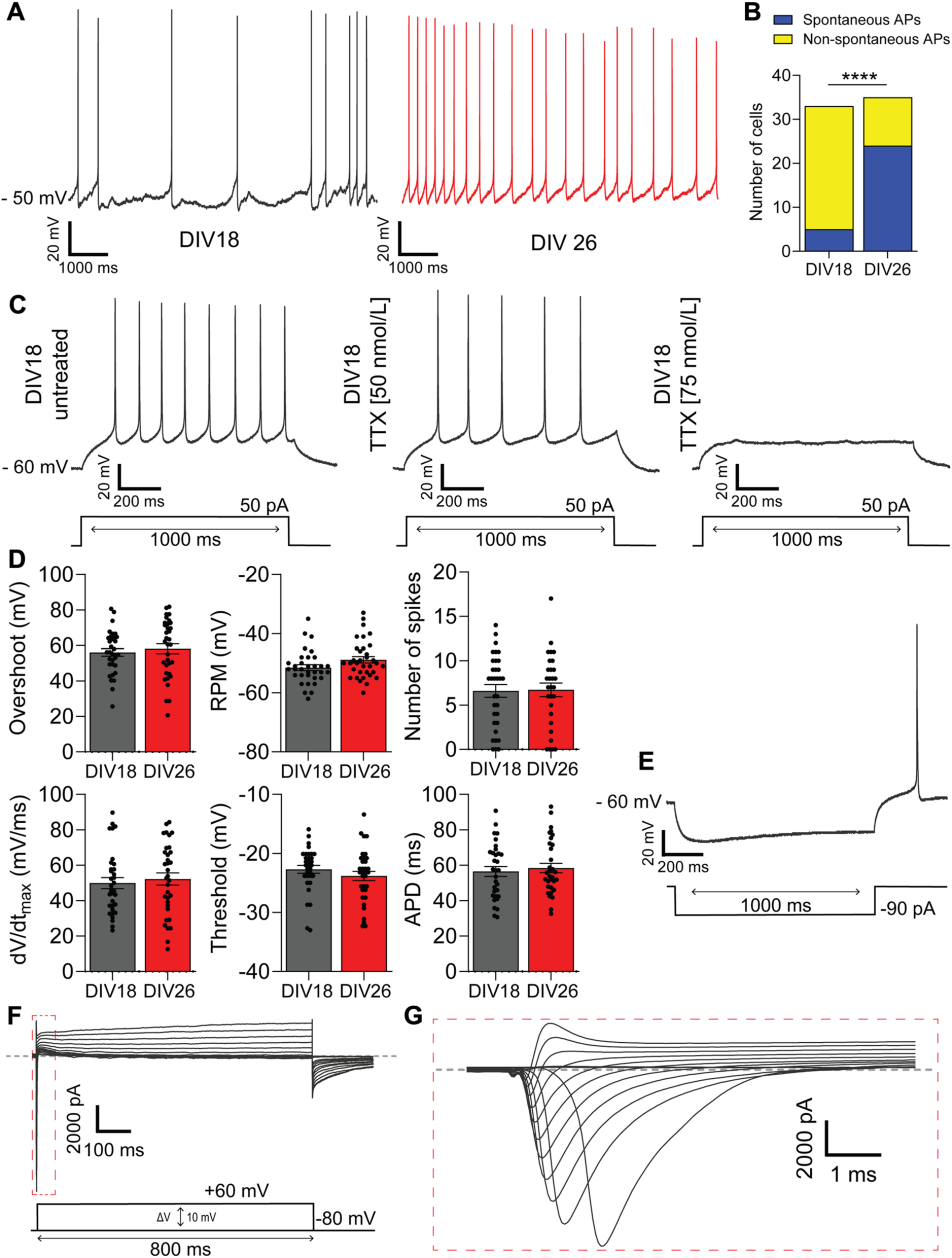
iDA neurons are electrically functional and mature at DIV 18. (**A**) Representative traces of the spontaneous action potentials (APs) recorded from DIV 18 (left, black) and the DIV 26 (right, red). (**B**) Stacked bar showing the cells number which fired spontaneous APs. Fisher’s exact test was used to assess significance within each group ****p < 0.0001. (**C**) Representative APs from DIV 18 in response to a 50 pA depolarizing current injection (bottom) before and after TTX treatment (50 and 75 nmol/L). (**D**) Bar graph showing the mean ± SEM of the resting potential membrane (RPM), the spikes frequency or number, the threshold, the overshoot, the dV/dtmax and the AP duration APD) from DIV 18 (n = 33 cells from n = 3 independent experiments) and DIV 26 (n = 34 cells from n = 3 independent experiments). A two-tailed unpaired Student’s t-test was used to assess differences between groups. (**E**) Representative AP from DIV 18 in response to a − 90 pA hyperpolarizing current injection. (**F**) Phase contrast image of a patched cell. Scale bar: 20 µm. (**G**) Representative voltage-activated Na^+^ and K^+^ currents elicited using 800 ms pulses from − 100 to + 60 mV in + 5 mV. The dashed line represents zero current. (**H**) Close-up view of voltage-activated Na^+^ current.

Our observation of iDA neurons’ capacity to synthesize and release DA and metabolites suggested that these cells could be electrically active and might reach maturity as early as DIV 18. To verify this hypothesis, we assessed some of the fundamental dopaminergic neuronal electrophysiological properties such as the ability of iDA neurons to fire action potentials (APs) using whole-cell patch clamp (Fig. 4). Importantly, electrical recordings of single cells showed that cells displayed spontaneous APs with a resting potential membrane (RPM) around -50 mV, consistent with characteristics of mature midbrain DA neurons^28^. Interestingly, more spontaneous APs bursts were observed at DIV 26 (15% and 68% at DIV 18 and DIV 26 respectively), suggesting that more functional synapses are present at the latest time point (Fig. 4A,B). Next, we tested the effect of Tetrodotoxin (TTX), a neurotoxin that specifically blocks voltage gated Na^+^ channels in DA neurons in vivo^29^. The spike frequency upon depolarizing current was reduced with the 50 nmol/L TTX treatment and was further reduced to zero with 75 nmol/L of TTX, suggesting that iDA neurons show the presence of active membrane events with functional inward sodium and outward potassium channels (Fig. 4C).

We further analysed key AP properties of the iDA neurons, such as the RPM, overshoot (corresponding to the amplitude above 0 mV), spike frequency, upstroke velocity (dV/dt_max_), AP threshold (determined at the first peak) and AP duration (APD; calculated at the threshold potential) (Fig. 4D). After hyperpolarizing current injection, 27% and 37% of cells triggered single AP at DIV 18 and DIV 26 respectively (Fig. S5A). Voltage-clamp recordings showed that at DIV 18, cells had fast gating inward currents and outward currents activated by voltage (Fig. 4F,G). Interestingly, no differences in iDA AP characteristics were observed between DIV 18 and DIV 26, suggesting that the electrical properties of the neurons reached a certain level of maturity as early as DIV 18. Collectively, our electrophysiological data confirmed that iDA exhibit electrical properties of mature DA neurons as early as DIV 18. Furthermore, ICC analysis of patched iDA cells stained with Neurobiotin®-488 tracer confirmed the midbrain DA neuronal identity with positive expression for TH (Fig. S5B,C). Therefore, our electrophysiological data further confirm the midbrain DA neuronal identity.

### Use of iDA neurons to model PD and identify neuroprotective compounds

**Figure 5:**
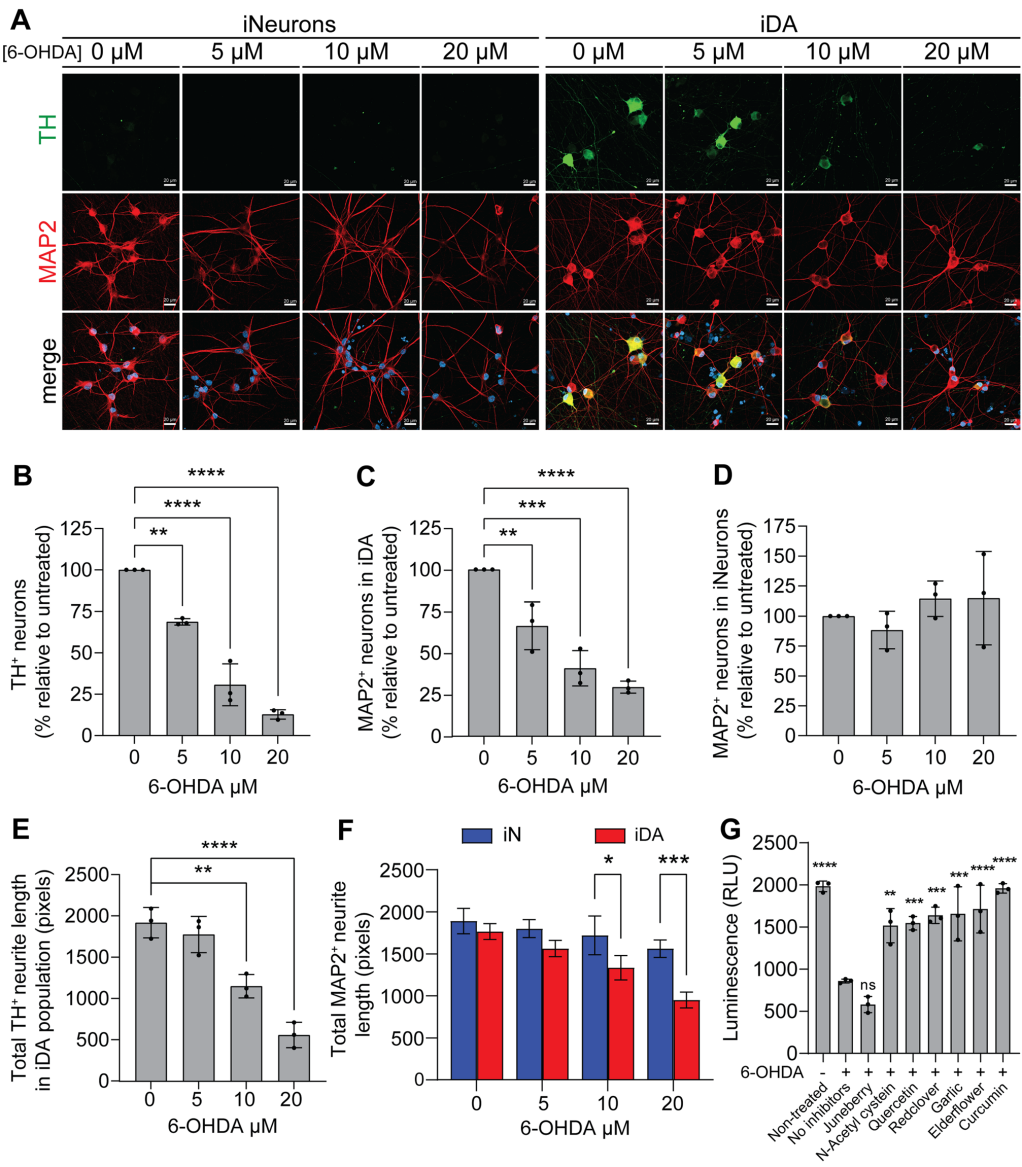
Treatment with 6-hydroxydopamine induces selective iDA neuronal loss. (**A**) Representative confocal images of ICCs against TH (green) and MAP2 (red) for iNeurons and iDA neurons showing selective loss of TH^+^ cells in iDA neuron culture, with increasing concentrations of 6-OHDA (5–10–20 µM) for 24 h (scale bar = 20 µm). (**B**) Quantitative analysis of the percentage of the TH^+^ neurons in iDA neuron culture, in comparison with the non-treated condition (n = 3). (**C**) Quantitative analysis of the percentage of MAP2^+^ cells in iDA neuronal culture, confirming the cell loss after 6-OHDA treatment (n = 3). (**D**) Quantitative analysis of the percentage of MAP2^+^ cells in iNeurons culture showing that 6-OHDA treatment had no effect on cell viability (n = 3). Statistical differences were assessed performing a one-way ANOVA Dunnett's multiple comparisons test comparing the mean of each treatment condition to the non-treated condition which is normalized to 100%. **p < 0.005; ***p < 0.0005; ****p < 0.0001. (**E**) Quantification of neurite length in iDA (TH^+^) neurons treated or non-treated with 6-OHDA for 24 h (n = 3). Statistical differences were assessed performing a one-way ANOVA Dunnett's multiple comparisons test comparing the mean of each treatment condition to the non-treated condition which is normalized to 100%. **p < 0.005; ***p < 0.0005; ****p < 0.0001. (**F**) Quantification of neurite length in iDA neurons and iNeurons (MAP2^+^) treated or non-treated with 6-OHDA for 24 h (n = 3). Statistical differences were assessed performing a 2-way ANOVA Šídák's multiple comparisons test comparing iDA neurons to iNeurons for each treatment condition. *p < 0.05; ***p < 0.0005. (**G**) iDA neuronal viability measured using CellTiter-Glo® Luminescent Cell Viability Assay. iDA neurons were pre-treated with indicated small molecules and then exposed to the 6-OHDA (20 µM) (n = 3). Statistical differences were assessed performing a one-way ANOVA Dunnett's multiple comparisons test comparing treated conditions to the control condition. ns = non-significant; ****p < 0.0001.

We next evaluated the potential use of the iDA neurons in a cellular assay to identify small molecules able to protect neurons against the toxicity 6-hydroxydopamine (6-OHDA), a molecule causing selective degeneration of DA neurons, likely through its uptake by the DAT^30–32^. First, we treated the iDA neurons at DIV 26 with increasing concentrations of 6-OHDA (5, 10, and 20 μM) for 24 h and then assessed the TH^+^ cell loss using ICC. Data showed that 6-OHDA treatment induced a TH^+^ neuronal loss in a concentration-dependent manner, with more than 80% of DA neuronal loss at the highest 6-OHDA concentration (Fig. 5A,B). Furthermore, the decrease of the proportion of MAP2^+^ cells confirmed that the loss of the TH^+^ signal is indeed due to neuronal death in iDA culture and not to a loss of the dopaminergic phenotype (Fig. 5C). Interestingly, no significant cell loss was detected in iNeurons culture, thus confirming the selective vulnerability of the iDA neurons to the 6-OHDA treatment (Fig. 5D). Of note, TH^+^ cell loss was associated with various apparent morphological characteristics of neuronal cell death, notably neurite fragmentation, as compared to non-treated iDA neurons or iNeurons (Fig. 5E,F). Then, we used iDA neurons to evaluate the potential neuroprotective effect of a series antioxidants small-molecules against 6-OHDA neurotoxicity. We assessed cellular viability by measuring the intracellular ATP levels using CellTiter-Glo® Cell Viability Assay. Prior to treatment with 6-OHDA, iDA neurons were incubated with several botanical extracts with high antioxidant capacities. Our results showed that pre-treatment with elderflower, red clover, curcumin, garlic, Quercetin and N-acetyl cysteine significantly reduced 6-OHDA-induced cell death, but not juneberry, thus confirming the previously reported neuroprotective capacities of these small-molecules^33–38^ (Fig. 5G). Collectively, our results confirm the utility of iDA neurons to model PD in culture and for the screening and the identification of neuroprotective compounds with a therapeutic potential for the treatment of PD and related disorders.

## Discussion

Understanding the mechanisms involved in the progression and pathogenesis of PD has motivated extensive research on devising methods for the development of functioning midbrain DA neurons from hiPSCs. These efforts exemplify hiPSCs as a valuable tool for investigating PD for future development of novel anti-parkinsonian drugs, and personalized therapies^4^. DA neurons are now one of the most studied brain-derived neuronal subtypes, and there has been a plethora of methods directed towards developing methods for the proper differentiation of hiPSCs into DA neurons. Several applications have demonstrated good efficiency in directing hiPSC differentiation into DA neurons. Nonetheless, there are major drawbacks from many of these recently published protocols lies in the significant time consumption for the generation of DA neurons (average of 40–90 days)^15,39–41^. Moreover, the proposed techniques appear to still be quite complex, since they are also dependent on defined culture media with sequential and precise chemical additions^15,42^, Transcription factor modifications^9,43^, the use of small-molecule inhibitors and activators^8,44^, or co-culturing with different cell types such as astrocytes^45^ or microglia^46^.

A recent approach by Zhang et al. have paved new roads in hiPSCs generated neurons by using what is now known as the *NGN2*-iNeuron protocol that can rapidly and reproducibly differentiate hiPSCs to different neural fates^16,19,23,47,48^. Here, we describe a improved method that makes use of the *NGN2*-iNeuron protocol for the patterning of hiPSC towards what we refer to here as iDA neurons. Indeed, our study shows that by using *NGN2* derived neurons combined with the simple use of commercially available midbrain defined differentiation and maturation media allows for the generation of a high (60%) homogenous population of midbrain DA neurons in a short lapse of time, with a minimum contamination by other brain cell types. These iDA neurons exhibit midbrain neuronal characteristics, can produce and release DA and are electrically and synaptically active as early as DIV 18.

It is known that DA neuron development and patterning is a highly coordinated process that requires the expression of specific transcription factors at different stages of its development^25,26,49,50^. In our study, the expression of the gene *LMX1a,* which is essential for the specification of midbrain DA neurons^51^, supports the timing of this articulated differentiation process in our iDA neurons. Likewise, our data showed a strong upregulation of the expression of the DA maturation TFs *NURR1, EN1*, and *PITX3*^52–54^, where we observe a significant increase in these gene expression levels between DIV18 and 26 of iDA maturation. Similarly, the expression of other developmental regulators like *FOXA2* were highly expressed in our iDA neurons^55^. Finally, multiple DA genes, related to neurotransmitter metabolism and signaling such as *DAT*, *D2R* and *VMAT2* were also found to be highly expressed in our iDA neurons^56–58^. Overall, our gene expression analysis confirms the successful induction and formation of DA neurons from the *NGN2*-iNeurons. Our gene expression analysis also revealed that iDA neurons exhibited the expression of two specific A9 DA neuron genes, namely *GIRK2,* and *ALDH1A1*^59^, thus confirming the capacity of our approach to generate specific DA neuronal populations relevant for the study of PD pathogenesis. It is important to mention that our approach helped generate region-specific and highly pure midbrain neurons, with negligeable contamination with other brain cell types, thus offering a suitable model for drug screening and a valuable tool to specifically study the human A9 DA neurons, the most vulnerable neuronal population in PD^26,27^.

Likewise, our functional assays that included DA release measurements using HPLC, followed by electrophysiological recordings, further demonstrated the efficiency of iDA neurons maturation. Our data suggest that the dopamine production machinery is well developed as early as DIV 18 and further improves at DIV 26. Most recent work examining the physiological phenotype of induced DA neurons do not report release of DA and the DA metabolite until 45 days of differentiation or later^10,60,61^, and DA detection at earlier time points is often recorded with the addition of the dopamine precursor L-DOPA^9,60–64^. This further validates that the iDA neurons not only show early release of DA (DIV 18), but also confirm their level of maturity and ability to synthesize and release DA, even in the absence of exogenous L-DOPA stimulation^63^. Midbrain DA neurons are well characterized as neurons capable of spontaneous discharge with membrane potentials averaging between − 60 and − 70 mV^65^ and these functional properties have been reported in hiPSC-derived midbrain DA neurons with recordings of APs averaging between − 30 and − 50 mV reported as late as 50 or 70-days post differentiation^60,62,66,67^. Results from our study indicate that iDA cells can trigger spontaneous activity and APs of (− 50 mV and − 60 mV) after hyperpolarization, and these recordings were observed as early as DIV 18 and appear to increase as cells mature further. Likewise, iDA cells generate potassium currents and TTX-sensitive sodium currents, driven by NaV1.2 sodium channels expressed in midbrain dopaminergic neurons^68^. Moreover, the ability to trigger spontaneous activity and AP after hyperpolarization, means that iDA cells expressed the hyperpolarization-activated, cyclic nucleotide-gated channels (HCN), similar to what was observed in the midbrain dopaminergic neurons in the mouse^69^. Together with the HPLC, These electrophysiology data support the in vitro generation of dopaminergic pace-making activity in our cultured iDA neurons.

Finally, 6-OHDA induced loss of dopamine (DA) neurons has helped serve as a method to produce an animal model of DA neuron loss in PD^70^. Therefore, work on developing a more rapid and equally efficient in vitro model with characteristics of selective DA neuron loss would be highly valuable to understand what triggers cell death in DA neurons and consequently help in identifying drugs that can counteract toxicity induced in these cells. In our study we confirmed that our protocol promotes the differentiation of mature iDA neurons that show selective sensitivity to 6-OHDA. Moreover, we demonstrated the utility of this model by examining potential small molecule drugs that can help promote survival and counteract toxicity induced by 6-OHDA.

In conclusion, our work introduces a highly robust optimized protocol for DA neuron differentiation from hiPSCs, which we believe has potential capabilities for studying the pathogenesis of PD in vitro. The DA differentiation protocol we designed here offers attractive prospects for accelerated specification of DA neurons while significantly reducing the expense of obtaining DA neurons of high purity. The strategy of implementing the use of *NGN2*-iNeuron model with an optimized easy to follow protocol of media maintenance appears to provide a high homogeneity population of iDA neurons in ranges higher than what has been previously published (as reviewed in^65^). Moreover, iDA neurons developed here exhibit molecular and functional characteristics, as well as the vulnerability to neurotoxin, of the ventral midbrain A9 subtype, and offer a viable cellular model to study PD pathogenesis. Finally, to make our approach suitable for other applications, including cell transplantation therapy, a further refinement of the differentiation protocol could be considered. Indeed, integration-free iPSC differentiation approach utilizing mRNA-based methods represents a valuable alternative to viral delivery system for an efficient and safe overexpression of *NGN2* in iPSC^10,71^. Likewise, the scalability of the differentiated iDA neurons, in addition to the feasibility of cryopreserving iDA precursors^71^ are aspects that can be explored to emphasize the value of the approach developed here. Collectively, such optimizations can present our model as a conventional method for clinical applications.

## Materials and methods

### Generation and maintenance of NGN2-hiPSCs

The parental AIW002-02 hiPSC line was described previously and analyzed for multiple quality control parameters, including pluripotency by immunofluorescence, trilineage differentiation potential and genomic integrity using the hiPSC Genetic Analysis Kit (Stemcell Technologies, 07550)^72^. AIW002-02 hiPSCs were split with ReLesR one day prior to simultaneous lentiviral transduction with two separate vectors encoding *NGN2* and rtTA and seeded at a density of 135,000 cells per 6-well in mTesR media (85850; Stemcell technologies, Vancouver, B.C, Canada) supplemented with Y27632 (S1049; Selleck Chemicals, Houston, TX, USA). Similarly, AIW002-02 hiPSCs were simultaneously transduced with the *NGN2*, rtTA and TMEM192-HA viruses. The media was changed to mTesR only one hour prior to transduction at a dilution factor of 1/100 for each virus, which yielded a calculated multiplicity of infection (MOI) of approximately 1 based on genomic qPCR analyses for woodchuck hepatitis virus post-transcriptional response element (WPRE) relative to albumin (Alb). The transduction was carried out in mTesR media plus 4 µg/ml of polybrene and was allowed to proceed for 24 h prior to puromycin selection at 1 µg/ml for 48 h, AIW002-02 hiPSCs were transduced with the *NGN2*, rtTA and TMEM192-HA viruses, cells were selected with puromycin and blasticidin at 1 µg/ml for 48 h. At this point of selection, the non-transduced control parental AIW002-02 cells did not survive. Throughout the transduction, selection and subsequent expansion steps, the media was changed daily. The AIW002-02 *NGN2* rtTA line was confirmed to be pluripotent by immunofluorescence for Nanog, Tra-1-80, Oct3/4 and SSEA, and are mycoplasma negative.

The two hiPSC cells stably expressing the doxycycline inducible *NGN2* cassette (AIW002-02 and AIW002-02-TMEM192-HA) were kindly provided by Dr. Thomas M. Durcan’s lab. For the maintenance of *NGN2*-hiPSCs, prior to differentiation, the two hiPSCs (AIW002-02 and AIW002-02-TMEM192-HA) stably expressing the doxycycline inducible *NGN2* cassette were maintained in mTesr™ Plus media (100-0276; Stemcell technologies, Vancouver, B.C, Canada) and seeded on Matrigel-coated plates (Matrigel Matrix hESC-qualified) (354277; ThermoFisher Scientific, Waltham, MA, USA). When hiPSC reached 70% confluency, they were split using gentle dissociation reagent (07174; Stemcell technologies, Vancouver, B.C, Canada). With each cell passage, mTesr™ Plus media was supplemented with ROCK Inhibitor Y27632 to enhance cell survival (S1049; Selleck Chemicals, Houston, TX, USA).

### Transduction of hiPSCs with lentivirus overexpressing NGN2

The following lentivirus vectors were used: 3rd generation lentiviral packaging plasmids pMDLg/pRRE (Plasmid 12251; Addgene Inc., Cambridge, MA) and (Plasmid 12253; Addgene Inc., Cambridge, MA), the VSV-G envelope expressing plasmid (Plasmid 12259; Addgene Inc., Cambridge, MA), in addition to the plasmids expressing the (FUW-TetO-*NGN2*-T2A-puromycin) and (FUW-Ubi-rtTA) which were a kind gift from Dr. Thomas C. Südhof lab^16,73^. Lentiviral vector stocks were generated by calcium phosphate-mediated transient transfection in HEK-293T cells (ATCC) as previously described (9,765,382). After 24, 48 and 72 h, lentiviral vector particles were harvested, and concentrated by ultracentrifugation. The viral titer was measured by QuickTiter™ Lentivirus Quantitation Kit (VPK-112; Cell biolabs, San Diego, CA).

The hiPSC line used for viral transduction is the parental ND36091 hiPSC line, this line was described previously^74^ and was analyzed for quality control parameters. Briefly, The ND36091 hiPSC line was produced in collaboration with the Tom and Sue Ellison Stem Cell Core (Institute for Stem Cell & Regenerative Medicine, University of Washington) via episomal reprogramming of human primary fibroblasts as described previously^75,76^. ND36091 hiPSC was split with gentle dissociation reagent (07174; Stemcell technologies, Vancouver, B.C, Canada) one day prior to simultaneous lentiviral transduction with the two separate vectors encoding *NGN2* and rtTA and seeded at a density of 150,000 cells per 60 mm plate in mTesR media (85850; Stemcell technologies, Vancouver, B.C, Canada) supplemented with Y27632 (S1049; Selleck Chemicals, Houston, TX, USA). Only virus preparations with > 80% infection efficiency as assessed by puromycin resistance at 4 µg/ml were used for experiments. The titer used for the transduction was the following: 2.97 × 10^9^ for the *NGN2* lentivirus vector and 1.34 × 10^9^ for the rtTA lentivirus vector. The transduction was carried out in mTesR media and was allowed to proceed for 24 h prior to starting further experimentation.

### Differentiation of NGN2-hiPSCs into iNeurons

For the differentiation to iNeurons of the two stably expressing *NGN2* hiPSCs lines (AIW002-02 and AIW002-02-TMEM192-HA), and the ND36091 hiPSC line overexpressing *NGN2*, we followed a previously published protocol by^16^ and optimized by The Early Drug Discovery Unit (EDDU) at McGill University. Briefly, one day after seeding (DIV 0), the media was switched to Day 0/1 medium (DMEM/F12 (10565042-018; ThermoFisher Scientific/Gibco, Waltham, MA USA), N2 (17504-044; ThermoFisher Scientific/Gibco, Waltham, MA USA), B27 (17502-048; ThermoFisher Scientific/Gibco, Waltham, MA, USA), NEAA (321–011-EL; Wisent Life Sciences, Quebec, QC, Canada), BDNF (450-02; Peprotech, Rocky Hill, NJ, USA), GDNF (450-10; Peprotech, Rocky Hill, NJ, USA), mouse laminin (23017015; ThermoFisher Scientific/Gibco, Waltham, MA, USA) and 2 µg/ml doxycycline (631311; Takara Bio Company, Mountain View, CA, USA) to induce *NGN2* expression. The next day (DIV 1), full media was changed for fresh Day 0/1 media supplemented with puromycin at 4 µg/ml for the ND36091 hiPSC line overexpressing *NGN2.* On day 2 (DIV 2), cells were dissociated with accutase (A1110501; ThermoFisher Scientific, Waltham, MA USA) and plated on coverslips coated with Poly-L-Ornithine at 50 µg/ml (P3655-10MG; Sigma-Aldrich, St. Louis, MO, USA) and laminin at 10 µg/ml. At this point the media was switched to Day 2 media (neurobasal medium (21103-049; ThermoFisher Scientific/Life Technologies, Waltham, MA, USA), N2, B27, GlutaMax (35050061; ThermoFisher Scientific, Waltham, MA, USA), NEAA, BDNF, GDNF, mouse laminin and 2 µg/ml doxycycline. iNeurons cells were maintained in Day2 media using long-term doxycycline induction while changing half of the media with fresh Day 2 every 2 days. From DIV 2 to DIV 4, Day 2 media was supplemented with Cytosine β-D-arabinofuranoside hydrochloride (Ara-C) at 2 µM to inhibit the proliferation of the remaining dividing cells (C6645-100MG; Sigma-Aldrich, St. Louis, MO, USA). Starting from DIV 5, iNeurons were washed with dPBS enriched in Ca^+^ and Mg^+^ to remove the cellular debris and the media was replaced with Day 2 media.

### Generation and culture of hiPSC-derived NPCs

The two NPC lines used in the study NPC-1 and NPC-2 were kindly provided by Dr. Aurélie de Rus Jacquet, and were derived from the two hiPSC lines (ND38554-obtained from National Institute of Neurological Disorders and Stroke (NINDS) Repository at the Coriell Institute for Medical Research) and (ND36091-described in^74^) respectively; and were both analyzed for quality control parameters. The step-by-step protocol for the differentiation of the iPSCs into midbrain floor-plate NPCs, and quality control experiments have been described in^77^. After the differentiation was complete at DIV 13, the cells were maintained in NPC medium (Neurobasal/B27 without vitamin A, 20 ng/ml FGF2, and 20 ng/mL EGF) for 7 days, the media was supplemented with ROCK inhibitor (Y-27632, Stemcell technologies, Vancouver, B.C, Canada) at each single-cell passage.

### Differentiation of NGN2-hiPSCs into iDA neurons

iNeurons from DIV 2 that were subjected to differentiation into dopaminergic (iDA) neurons (iDA-1 derived from stable AIW002-02 hiPSC expressing *NGN2* and iDA-2 derived from stable AIW002-02-TMEM192-HA hiPSC expressing *NGN2*) and (iDA-3 derived from ND36091 hiPSC viraly overexpressing *NGN2*) by first exposing the cells to Differentiation media from STEMdiff™ Midbrain Neuron Differentiation Kit (100-0038; Stemcell technologies, Vancouver, B.C, Canada) supplemented with 2 µg/ml doxycycline and Human Recombinant SHH at 200 ng/ml. From DIV 2 to DIV 4, the media was fully changed every day and supplemented with Ara-C at 2 µM. From DIV 5 to DIV 8, half of the media was changed every day. Starting from Day 9 (DIV 9), the media was fully replaced with midbrain Maturation media obtained from STEMdiff™ Midbrain Neuron Maturation Kit (100-0041; Stemcell technologies, Vancouver, B.C, Canada) supplemented with 2 µg/ml doxycycline. Starting from Day 10 (DIV 10) and every two days, half of the media was replaced with fresh Maturation media.

### Differentiation of hiPSC-derived NPCs into iDA neurons

NPCs from DIV 18 (corresponding to DIV 2 for iDA neurons), were subjected to differentiation into dopaminergic neurons by first exposing the cells to Differentiation media from STEMdiff™ Midbrain Neuron Differentiation Kit (100-0038; Stemcell technologies, Vancouver, B.C, Canada) supplemented with Human Recombinant SHH at 200 ng/ml. Starting from DIV 25 (corresponding to DIV 2 for iDA neurons), the media was fully replaced with midbrain Maturation media obtained from STEMdiff™ Midbrain Neuron Maturation Kit (100-0041; Stemcell technologies, Vancouver, B.C, Canada). Starting from DIV 26 (corresponding to DIV 10 for iDA neurons) and every two days, half of the media was replaced with fresh Maturation media.

### Immunocytochemistry and quantification

At each time point, iNeurons and iDA were fixed using with cold 4% paraformaldehyde (PFA), 2% sucrose for 15 min at room temperature. PFA was then washed 3 times with PBS for 5 min each. Cells were then permeabilized with 0.3% Triton X-100/PBS (T8787-100ML; Sigma-Aldrich, St. Louis, MO, USA) for 30 min at 4 °C. Then, cells were blocked for 30 min at room temperature (RT) in a blocking buffer (5% Normal Goat Serum, 1% BSA, 0.3% Triton X-100/PBS). Primary antibodies were added in the blocking buffer and incubated overnight at 4 °C. After three washes in PBS 0.3% Triton X-100 of 10 min each, secondary antibody conjugated with Alexa Fluor 488 or 594 (Invitrogen, Waltham, MA, USA) was added and incubated for 2 h at RT. After three washes in PBS 0.3% Triton X-100 of 10 min each, the cells were then incubated with 4′,6-diamidino-2-phenylindole (DAPI) nuclear counterstain for 2 min at 1:5000. After a last wash with PBS, the coverslips were mounted with Fluoromount-G™ (17984-25; Electron Microscopy Sciences, Hatfield, PA, USA) and imaged using a Zeiss LSM800 confocal microscope (Zeiss, Oberkochen, Germany). Refer to Table S1 for detailed description of antibodies, and the dilutions used in the study.

Quantification of TH^+^ cells using Fiji software^79^. Briefly, TH^+^ cells were counted and divided by either the total number of cells (DAPI counting), MAP2^+^ cells or β-III-tubulin^+^ cells. The quantification was made for four independent experiments and was performed by two experimenters.

### RNA isolation and gene expression analysis

Total RNA was extracted from cells during differentiation at DIV2, 6, 10, 14, 18, 22 and 26 for both iNeurons and iDA neurons using the RNeasy Mini Kit (74104; Qiagen, Hilden, Germany). RNA was quantified, and for each sample 1 μg of total RNA was treated with ezDNase™ Enzyme (18091150-ThermoFisher Scientific, Waltham, MA, USA), and reverse-transcribed using SuperScript™ IV First-Strand Synthesis System from ThermoFisher Scientific (18091150-ThermoFisher Scientific, Waltham, MA, USA) according to the manufacturer’s protocol.

Quantitative RT-PCR (qRT-PCR) was performed using the QuantiNova SYBR Green PCR Kit using 10 μg/ml of cDNA according to the manufacturer’s protocol (208054; Qiagen, Hilden, Germany), and run on The LightCycler® 96 Instrument (Roche Diagnostics) under the following conditions: PCR initial activation step 2 min at 95 °C, 2-step cycling denaturation 5 s at 95 °C, and combined annealing and extension for 10 s at 60 °C, performed for 40 cycles. Transcript levels of GAPDH were measured as endogenous controls. All qRT-PCR experiments were performed in triplicates. Gene expression was analyzed based on the delta CT (ΔCT) approach and normalized to the expression of GAPDH relative to the control at DIV2. Gene expression analysis were also presented as the mean of the raw CT values at DIV26. Primer sequences, annealing temperatures, and PCR product sizes are listed in Supplementary Table S2.

### SDS-PAGE and Western blot analysis

Total protein fraction was extracted by dissolving the cell pellet in 2 × Laemmli buffer (0.125 M Tris–HCl (pH 6.8), 20% glycerol, 0.2% 2-mercaptoethanol, 0.004% bromphenol blue, 4% SDS) and incubated at 95 °C for 20 min. Five µl of the total protein fraction (corresponding to 25–35 µg of proteins) was loaded per well in a 10% SDS-PAGE gel. Gels were run at 160 V for 75 min, and the proteins were transferred to nitrocellulose membranes using the Trans-Blot Turbo™ Transfer system (Bio-Rad, Hercules, CA, USA). The membranes were incubated in blocking buffer (3% Fish Gelatin in PBS-Tween 0.1% (PBS-T) at RT for 1 h prior to O.N incubation with primary antibodies in the blocking solution (see Table S1 for antibody details). Membranes were then washed 3 times with PBS-T (10 min), incubated with the appropriate secondary antibodies, either 680RD-conjugated or 800 W-conjugated (LI-COR Lincoln, NE, USA) (see Table S1) and finally washed 3 times (10 min) with PBS-T. Visualization and quantification were carried out with the LI-COR Odyssey scanner and software (LI-COR Lincoln, NE, USA). At least three independent experiments were analyzed for SDS-PAGE.

### High-performance liquid chromatography (HPLC)

To determine the dopamine levels in the iDA neurons, we followed a protocol adapted from previously published work^78,80^). iNeurons and iDA neurons were plated in 12-well plates and exposed to the same conditions described earlier. At DIV 18 and 26 the media was collected and coupled with 14 µl of perchloric acid (7 M; Sigma-Aldrich: 244252), and the neurons were then washed with HBSS (Hanks' Balanced Salt Solution; Sigma-Aldrich: 55037C). The neurons were then incubated in HBSS with or without 56 mM of KCl (500 µl per well) for 30 min, and then the culture medium was collected and mixed with 7 μl of perchloric acid (7 M) and centrifuged at 4 °C. Supernatants were stored frozen at − 80 °C until assayed. To measure the intracellular catecholamine content, differentiated cells were lysed using a solution of 0.2 M perchloric acid (PCA) in PBS (100 µl per well) and collected with a cell scraper. Cell lysates were centrifuged at 4 °C and supernatants were stored frozen at − 80 °C until assayed. High-performance liquid chromatography (HPLC) was performed on lysates and culture media from iNeurons and iDA extracted with perchloric acid. The mobile phase consisted of 47.8 mM NaH2PO4, 0.9 mM sodium octyl sulfate, 0.4 mM EDTA, 2 mM NaCl, and 8% methanol at pH 2.9 and was delivered at 0.9 ml/min at 3200 psi using a 1525 Pump (Waters), as described previously. Neurotransmitters were separated with an Atlantis dC18 (3 μm) column kept at 28 °C (Waters) and quantified with a 2465 Electrochemical Detector in DC mode (range 5 nA, Ec + 0.7 V) linked to a chart recorder. DA, L-DOPA, norepinephrine, 3,4-dihydroxyphenylacetic acid (DOPAC), homovanillic acid (HVA), 5-HIAA, 3-MT and serotonin were used as standards for quantification and to identify which neurotransmitters and metabolites were produced in vitro.

### Electrophysiological study

Electrophysiological study was performed on iNeurons and iDA neurons on DIV 18 and DIV 26 according to a protocol previously described^81^. Patch-clamp experiments were performed at RT using an Axopatch 200B amplified and pClamp software v10 (Molecular Devices, CA, USA). Signals were filtered at 5 kHz, digitized at 10 kHz, and stored on a microcomputer equipped with an AD converter (Digidata 1440A, Molecular Devices). P/4 leak subtraction was used prior to applying pulse stimulation. Action potentials and macroscopic currents were recorded using the whole-cell configuration of the patch clamp technique in current- and voltage-clamp modes, respectively. The pipettes were made from borosilicate glass capillaries (Sutter Instrument, CA, USA) and were fire polished.

For the current-clamp experiments, the pipettes (resistance 6–20 MΩ) were filled with an intracellular solution containing (in mmol/L): 130 K-gluconate, 10 di-Na-phosphocreatine, 10 HEPES, 2 MgCl2, 2 Mg-ATP, and 0.4 GTP. The pH was adjusted to 7.3 with 1 N KOH. Neurobiotin (0.5%, SP-1125-2, Vector Laboratories, CA) was added to mark the recorded neuron for later immunochemistry. The external solution was composed of (in mmol/L): 154 NaCl, 5.6 KCl, 2 CaCl2, 1 MgCl2, 8 D-glucose, and 10 HEPES. The pH was adjusted to 7.3 with NaOH. The gap-free mode was used to record spontaneous APs. Then, the membrane voltage was maintained at − 60 mV. Action potentials (APs) were elicited using the following current step procedure: 0 pA for 200 ms followed by 10 pA steps ranging from 0 to 120 pA for 1000 ms. A hyperpolarizing current was injected by 10 pA steps ranging from 0 to − 120 pA. Tetrodotoxin (TTX) was used at 50 and 75 nmol/L (LATOXAN, France).

For the voltage-clamp experiments, the pipettes (resistance 2–3 MΩ) were coated with HIPEC (Dow-Corning, MI, USA) to minimize electrode capacitance and as previously described (PMID: 18337362). Series resistance and cell capacitance were corrected. The currents were obtained using 800 ms pulses from − 80 to + 60 mV in + 5 mV increment.

### 6-OHDA treatment

400 μM of 6-OHDA stock was prepared fresh by solubilizing 6-OHDA powder (162,957; Sigma-Aldrich, St. Louis, MO, USA) in filtered 0.02% l-ascorbic acid (A7506; Sigma-Aldrich, St. Louis, MO, USA). Varying concentrations of 6-OHDA (5, 10, and 20 μM) were prepared in the neuronal culture media and cells were treated for 24 h. Cells were then fixed with cold 4% PFA, 2% sucrose for 15 min at room temperature for immunofluorescence analysis. ICC was performed as described in the materials and methods section. Neurite length (measured in pixels) was quantified using the high content analysis software (HCA-Vision V2.2.0. CSIRO), neurite length measures the sum of the length of each neurite segment assigned to each neuron, and data are presented as the average per field acquired.

### Assessment of the neuroprotective activity of natural extracts against 6-OHDA treatment

iDA-1 neurons were plated in triplicates in white 96-well plates with optical bottom and incubated, at DIV-26, in the absence or presence of botanical extracts (elderflower (10 µM), red clover (20 µM), juneberry (10 µM), garlic (20 µM) and curcumin (10 µM)) for 72 h. The botanical extracts of elderflower, redclover, juneberry and garlic were provided by Dr. Aurélie de Rus Jacquet and were prepared as described in^35^. Curcumin was purchased from (C1386-10G; Sigma-Aldrich, St. Louis, MO, USA). After treatment with the botanicals, as a positive control, wells were reserved for incubation in the absence or presence of the two commonly used antioxidants Quercetin (0.1 µM)^82^ (Q4951-10G; Sigma-Aldrich, St. Louis, MO, USA) and N-acetyl cysteine (5 mM)^83^ (A7250-10G; Sigma-Aldrich, St. Louis, MO, USA) for 4 h. Next, the iDA-1 cells were incubated in fresh media containing 6-OHDA (20 µM) in the absence or presence of the extracts for another 24 h. Next, the intracellular ATP level was estimated using CellTiter-Glo® Luminescent Cell Viability Assay, an assay based on the enzymatic and luminescent transformation of luciferin to oxyluciferin in the presence of ATP (G7570; Promega, Madison, WI, USA). Briefly, 100 μL of CellTiter-Glo® Reagent was added to each well after the 24 h treatment with 6-OHDA and mixed for 2 min on an orbital shaker to promote cell lysis. The plate was then incubated for an additional 10 min and the luminescent signal was recorded using a Cytation 5 microplate reader (Biotek, Winooski, VT). At least three independent experiments were analyzed for CellTiter-Glo® Luminescent Cell Viability Assay.

### Statistical analysis

For immunocytochemistry, qRT-PCR, Western blot, and 6-OHDA experiments statistical significance was assessed performing a 2-way ANOVA Šídák's multiple comparisons test comparing iDA cells to iNeurons at each time point or each condition using Prism 9.0.0 (GraphPad, La Jolla, CA, USA). For electrophysiology experiments, all statistical analyses were performed using Prism 8.0 software. The normality of distribution was determined using D’Agostino-Pearson normality test. The data are expressed as mean ± SEM (standard error of the mean). A two-tailed unpaired Student’s t-test and a Fisher’s exact test were used. All the statistical tests were performed using a 95% confidence interval and the differences were considered significant beyond the risk threshold 0.05% (*p < 0.05, **p < 0.01, ***p < 0.001, ****p < 0.0001).

## Supplementary Information

Supplementary Information 1.

Supplementary Information 2.

Supplementary Information 3.

Supplementary Information 4.

Supplementary Information 5.

Supplementary Information 6.

Supplementary Information 7.

## Data Availability

The datasets used and/or analysed during the current study available from the corresponding author on reasonable request.
