# Supplementary material for: Combining NGN2 programming and dopaminergic patterning for a rapid and efficient generation of hiPSC-derived midbrain neurons
Source: Sci Rep. 2022 Oct 13;12:17176. doi: 10.1038/s41598-022-22158-4 (PMC9562300; doi:10.1038/s41598-022-22158-4)
Supplement: Supplementary file 1 — Supplementary Information 1. [file 41598_2022_22158_MOESM1_ESM.docx]

**Supplemental figure legends**

**Figure S1:** **Combination of** ***NGN2* overexpression and midbrain differentiation and maturation medias force the dopaminergic phenotype of the hiPSC-derived iNeurons.**

**(A**) Representative confocal illustrations of ICCs against TH (green) and βIII-Tubulin (red) for iNeurons or iDA neurons from 3 iPSC lines (iDA-1, iDA-2 and iDA-3) at different timepoints. Scale bars: 20 µm. (**B**) Quantitative analysis of the percentage of TH^+^ neurons for iNeurons and iDA-1, iDA-2 and iDA-3 neurons at different timepoints. Data represent the average percentage of TH^+^ neurons normalized against βIII-Tubulin^+^ neurons (n=4). Statistical differences were assessed performing a 2-way ANOVA Šídák's multiple comparisons test comparing iDA neurons to iNeurons at each time point. ****p* < 0.001; *****p* < 0.0001.

**Figure S2**: **Combination of** ***NGN2* overexpression and midbrain differentiation and maturation medias did not induce dopaminergic phenotype in neuronal progenitor cells.**

(**A**) Schematic timeline of the use of different conditioning media to differentiate neuronal progenitor cells (NPC) into dopaminergic neurons. NPCs midbrain cells are patterned from hiPSCs from DIV 0 to DIV 13 using media as described previously. Starting from DIV 11 to DIV 18, NPCs are grown in NPC media containing recombinant human FGFb and EGF for the successful generation of midbrain‐patterned NPCs. To induce the dopaminergic phenotype, the midbrain pattered NPCs are subjected to the same differentiation/maturation protocol used for iNeurons. NPCs are exposed to the midbrain differentiating media supplemented with SHH from DIV 18 (corresponding to DIV 2 for iDA neurons) until DIV 25 (corresponding to DIV 9 for iDA neurons). Differentiated iDA neurons are then exposed to maturation media allowing them to reach a late maturation stage at DIV 42 (corresponding to DIV 26 for iDA neurons). (**B**) Representative confocal illustrations of ICCs against TH (green), MAP2 (white) and βIII-Tubulin (red) for 2 NPC lines (NPC-1 and NPC-2) at different timepoints. Scale bars: 20 µm. (**C**) Quantitative analysis of the percentage of TH^+^ neurons for NPC-1 and NPC-2 at different timepoints. Data represent the average percentage of TH^+^ neurons normalized against MAP2^+^, βIII-Tubulin^+^ neurons or total number of DAPI nuclei (n=3).

**Figure S3: iDA neuronal culture presents a low contamination with other brain cell types.**

(**A**) Representative confocal illustrations of ICCs against TH, glial markers GFAP or Iba1, glutamatergic neuronal marker (VGlut1) and serotoninergic neuronal marker (SERT). (**B**) Quantitative analysis of the percentage of Iba1, GFAP, VGlut1 and SERT-positive cells in iDA culture.

**Figure S4: iDA neurons express key dopaminergic genes and related proteins**

(**A**) Whole sample quantitative qRT-PCR analysis of the expression levels of the key dopaminergic genes. (**B**) Comparison of gene expression profile in iNeurons, iDA-1 and iDA-2 neurons and NPC-1 and NPC-2 at DIV 26. The plot represents average CT values of the genes with a cut-off of 40 cycles (n=3). (**C**) Western blot analysis of the expression of key dopaminergic proteins markers at different time points.

**Figure S5: Correlation of electrophysiological assays with TH^+^ profiles in iDA neurons**. (**A**) Quantification of the proportion of cells triggering single AP after hyperpolarizing current injection. (**B**) Image of a patch pipette in contact with a neuronal membrane (Scale bar = 20µm). (**C**) Representative phase contrast images of iDA cells patched and stained with Neurobiotin®-488 tracer were correlated with confocal images with TH^+^ staining (scale bar = 25 µm). Corresponding electrophysiological readouts showing iDA properties are displayed below the images (red square for top cell, blue square for bottom cell) (Scale bar = 25 µm).

**Tables**

**Table S1:** List of the antibodies used in the present study.

| **Antigen/species** | **Clone name/catalog number** | **Epitope** | **Concentration (WB)** | **Concentration (ICC)** | **Source** |
| --- | --- | --- | --- | --- | --- |
| **Primary Antibodies** | | | | | |
| TH/mouse | LNC1/MAB318 | Outside of the regulatory N-terminus | 1:1000 | 1:1000 | Millipore Sigma |
| MAP2/rabbit | MAP2 antibody/4542S | Carboxy-terminal residues of human MAP2 | 1:1000 | 1:1000 | Cell Signaling Technology |
| MAP2/chicken | MAP2 antibody /PA1-10005 | Amino acids 235-1588 | N/A | 1:1000 | Invitrogen |
| TUJ1/rabbit | Anti-beta III Tubulin antibody/ab18207 | Proprietary (Abcam) | N/A | 1:1000 | Abcam |
| VGlut1/rabbit | Anti-VGLUT1 antibody/ V0389 | C-terminus of human VGLUT1 (amino aicds 504-520). | N/A | 1:300 | Millipore Sigma |
| Serotonin transporter/rabbit | Serotonin transporter antibody/ A19110 | amino acids 1-100 | N/A | 1:100 | Abclonal |
| GFAP/rabbit | Anti-Glial Fibrillary Acidic Protein antibody/G4546 | C-terminal sequence of human GFAP | N/A | 1:500 | Millipore Sigma |
| Iba1/rabbit | Anti Iba1 antibody/ 019-19741 | Iba1 C-terminal sequence | N/A | 1:1000 | FUJIFILM Wako |
| FoxA2/rabbit | FoxA2/HNF3β (D56D6) XP® antibody/8186 | Unspecified | 1:1000 | N/A | Cell Signaling Technology |
| PITX3/rabbit | PITX3 antibody/ 38-2850 | N-terminal region of the mouse PiTX3 protein | 1:1000 | N/A | Invitrogen |
| EN1/rabbit | EN1/Engrailed 1 antibody/ BS-11744R | amino acids 351-392 | 1:1000 | N/A | Bioss |
| LMX-1/rabbit | LMX-1 antibody/ AB10533 | C-terminus of LMX-1 | 1:1000 | N/A | Millipore Sigma |
| Actin | β-actin clone BA3R / G043 | Beta-actin N-terminal peptide-KLH conjugates. | 1:10,000 | N/A | Abm |
| **Secondary Antibodies** | | | | | |
| Alexa Fluor 488 goat anti-mouse (H+L) | Alexa Fluor 488 goat anti-mouse/A11029 | N/A | N/A | 1:400 | Invitrogen |
| Alexa Fluor 594 goat anti-rabbit (H+L) | Alexa Fluor 594 goat anti-rabbit/A11037 | N/A | N/A | 1:400 | Invitrogen |
| IRDye® 680RD Goat anti-Rabbit IgG Secondary Antibody | 680RD-conjugated goat anti-rabbit/ 926-68071 | N/A | 1:20000 | N/A | LI-COR Biosciences |
| IRDye® 800CW Goat anti-Rabbit IgG Secondary Antibody | 800CW-conjugated goat anti-rabbit/ 926-32211 | N/A | 1:20000 | N/A | LI-COR Biosciences |
| IRDye® 680RD Goat anti-Mouse IgG Secondary Antibody | 680RD-conjugated goat anti-mouse/ 926-68070 | N/A | 1:20000 | N/A | LI-COR Biosciences |
| IRDye® 800CW Goat anti-Mouse IgG Secondary Antibody | 800CW-conjugated goat anti-mouse/ 926-32210 | N/A | 1:20000 | N/A | LI-COR Biosciences |

**Table S2:** qRT-PCR primer design

| **GENE** | **Forward 5'-3'** | **Reverse 5'-3'** | **Product size** | **TA (°C)** |
| --- | --- | --- | --- | --- |
| ***ALDH1A1*** | CGGGAAAAGCAATCTGAAGAGGG | GATGCGGCTATACAACACTGGC | 267 | 60 |
| ***D2R*** | GAAAGCCACTCAGATGCTCG | GCAGTGGAGGATCTTCAGGA | 223 | 60 |
| ***DAT*** | TCACCACCTCCATCAACTCC | GGGTGAGCAGCATGATGAAG | 210 | 59 |
| ***EN1*** | GTGGTCAAAACTGACTCGCAGC | CCGCTTGTCCTCCTTCTCGTTC | 235 | 60 |
| ***FOXA2*** | ACAACCTCATGTCCTCGGAG | TCATAATGGGCCGGGAGTAC | 228 | 59 |
| ***GAPDH*** | AGGTCGGAGTCAACGGATTT | ATCTCGCTCCTGGAAGATGG | 230 | 60 |
| ***GIRK2*** | GGTCTTTTCCACCCATGCAG | ACACCAGAAACAGACGGTCA | 215 | 59 |
| ***LMX1A*** | CTCAACAGAGGCGAGCATTC | GGCCAGCTTCTTCATCTTCG | 152 | 59 |
| ***MAP2*** | CCCCTTGCTTCCCTGTAGAA | ATTTCCTCCTGGCAACCTCA | 231 | 59 |
| ***NURR1*** | CACTTCTCTCCCCAGCTTCA | CGGCATCATCTCCTCAGACT | 184 | 59 |
| ***OCT4*** | CTCTGAGGTGTGGGGGATT | AGCTAAGCTGCAGAGCCTCA | 419 | 64 |
| ***PTX3*** | CAGGCCGAGCTATGCAAAG | GACCGAGTTGAAGGCGAATG | 159 | 59 |
| ***SOX2*** | ACTTTTGTCGGAGACGGAGA | TATAATCCGGGTGCTCCTTC | 86 | 64 |
| ***TH*** | CCGAGCTGTGAAGGTGTTTG | ACACTTGTCCAGCTCTGACA | 238 | 59 |
