# Supplementary figures and images for: Combining NGN2 programming and dopaminergic patterning for a rapid and efficient generation of hiPSC-derived midbrain neurons
Source: Sci Rep. 2022 Oct 13;12:17176. doi: 10.1038/s41598-022-22158-4 (PMC9562300; doi:10.1038/s41598-022-22158-4)

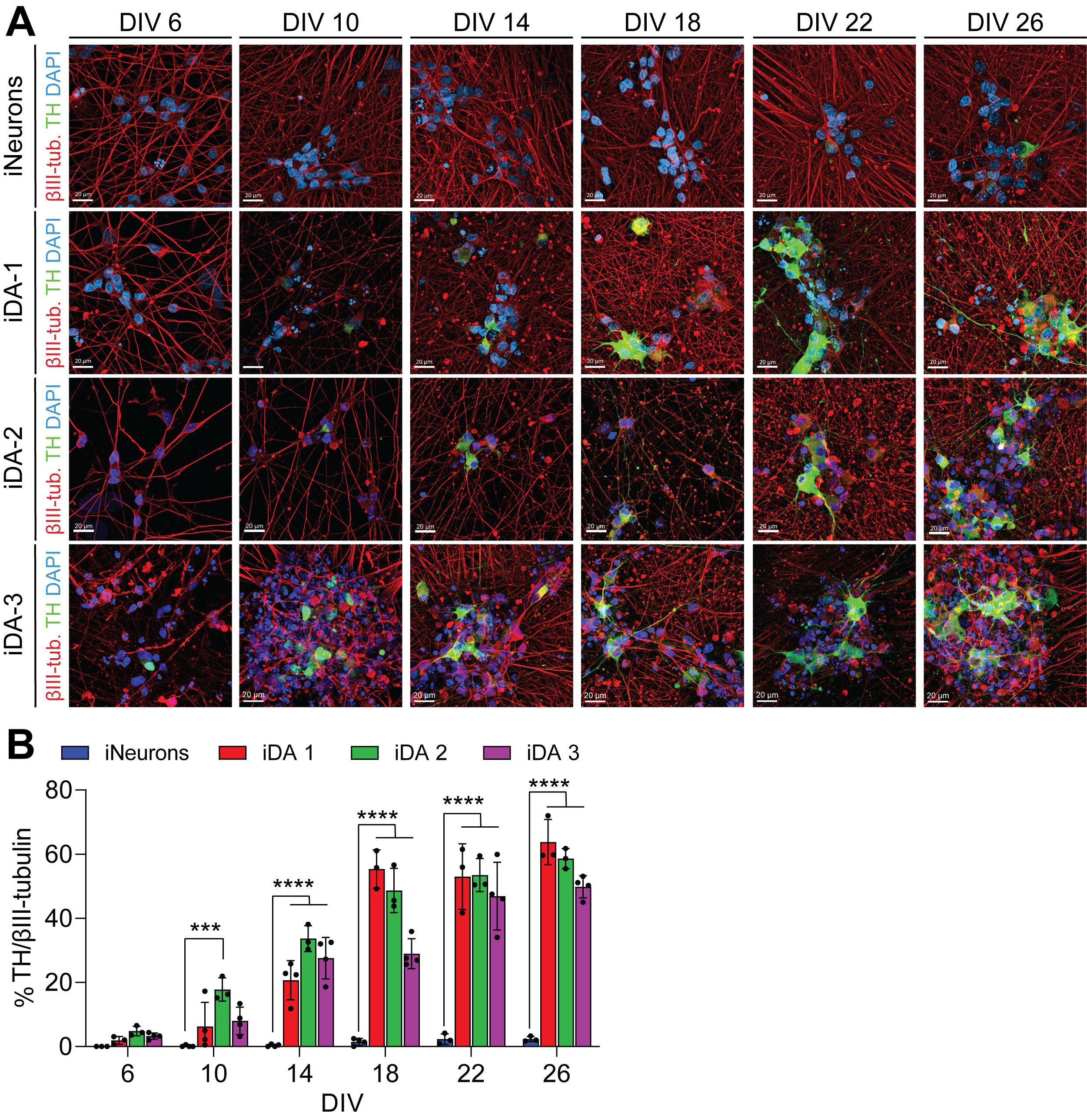

Supplement: Supplementary file 2 — Supplementary Information 2. [file 41598_2022_22158_MOESM2_ESM.tif]

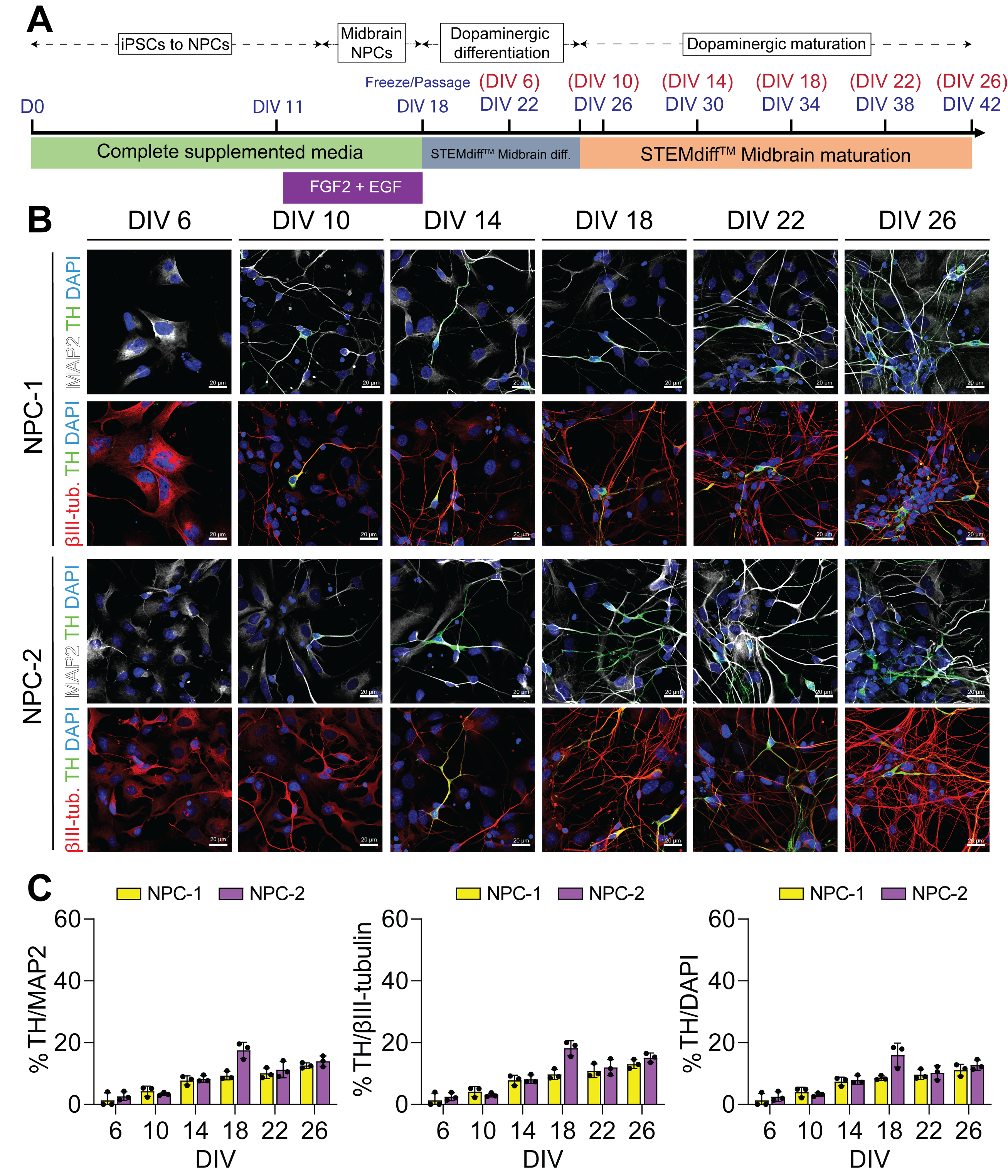

Supplement: Supplementary file 3 — Supplementary Information 3. [file 41598_2022_22158_MOESM3_ESM.tif]

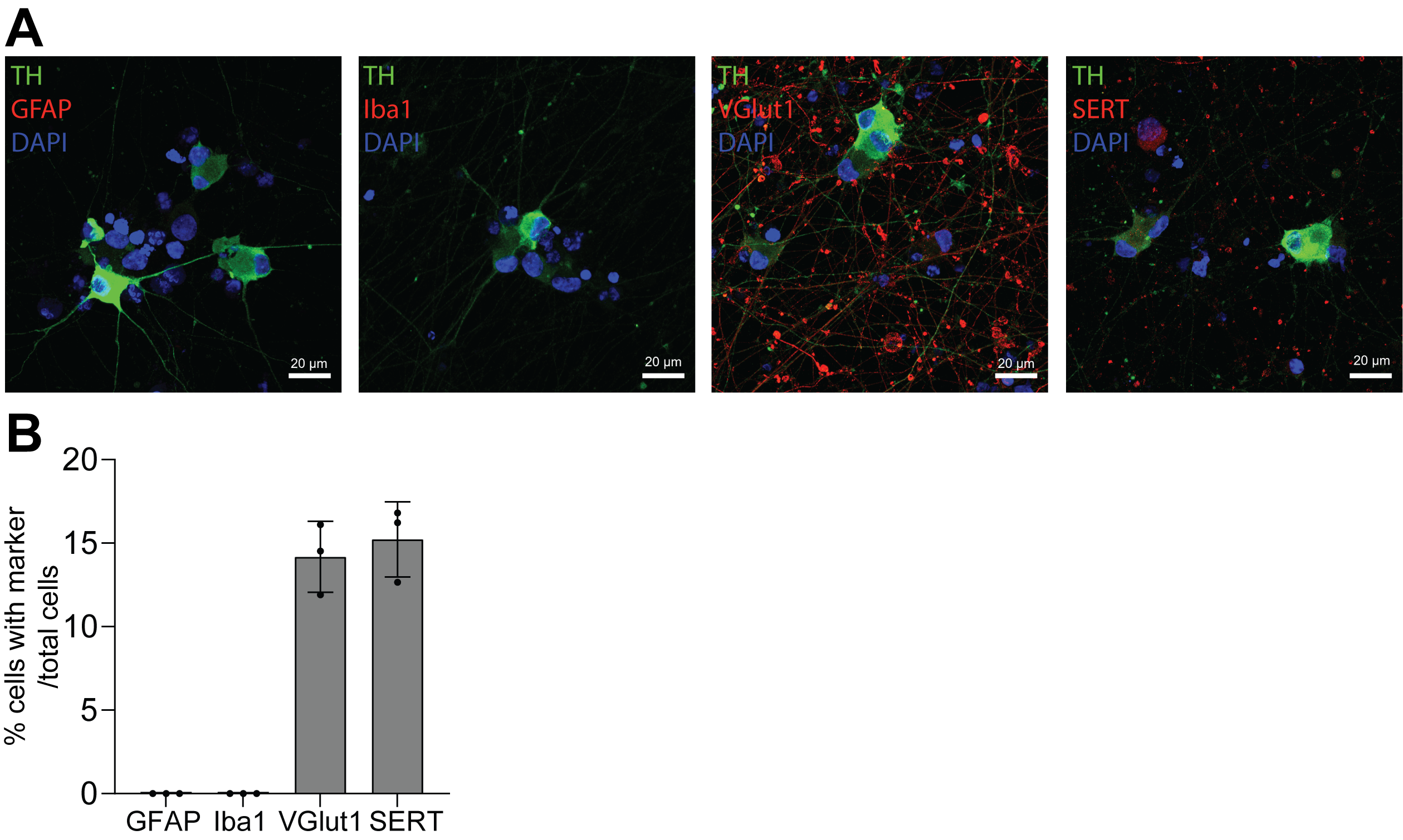

Supplement: Supplementary file 4 — Supplementary Information 4. [file 41598_2022_22158_MOESM4_ESM.tif]

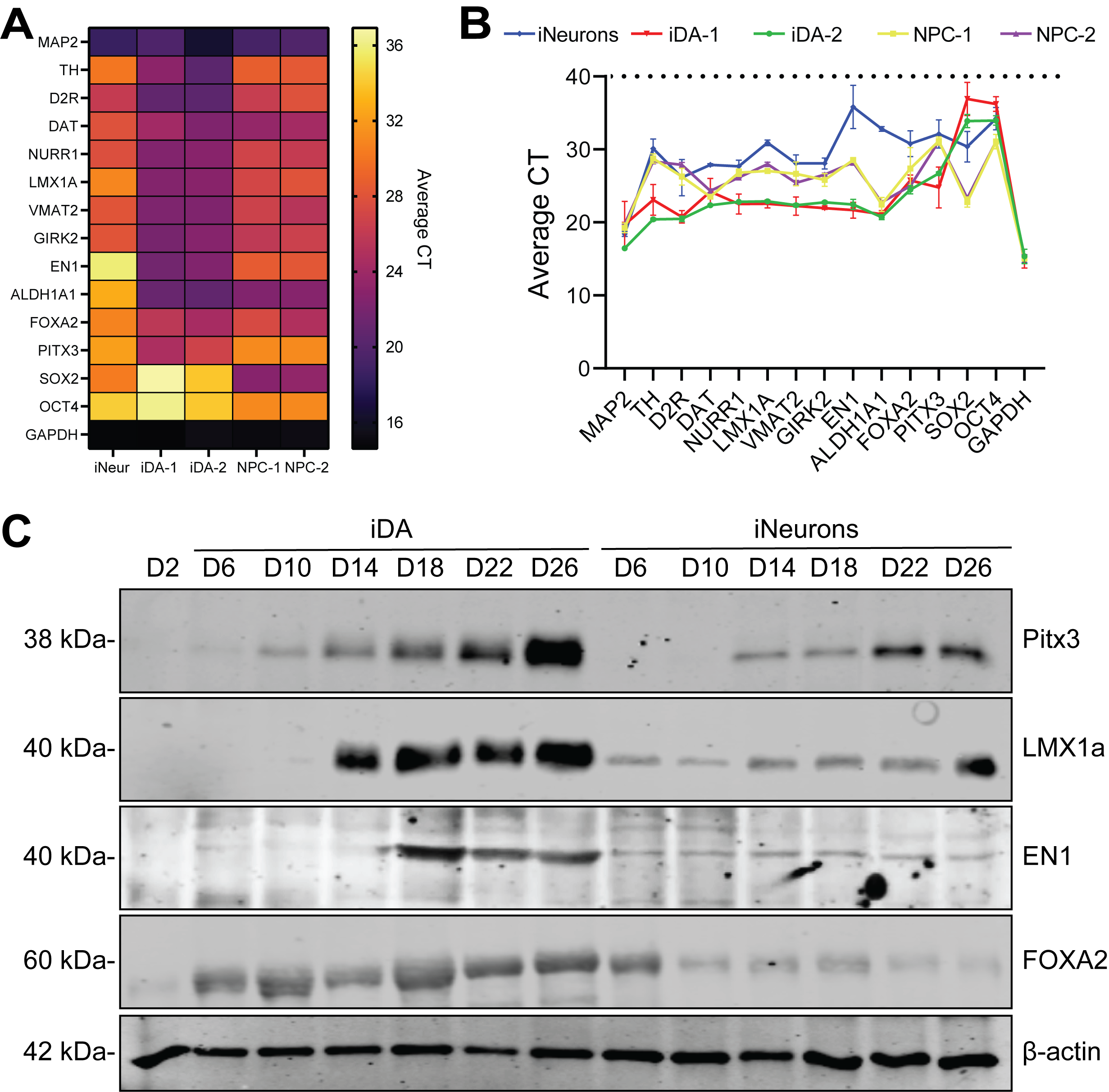

Supplement: Supplementary file 5 — Supplementary Information 5. [file 41598_2022_22158_MOESM5_ESM.tif]

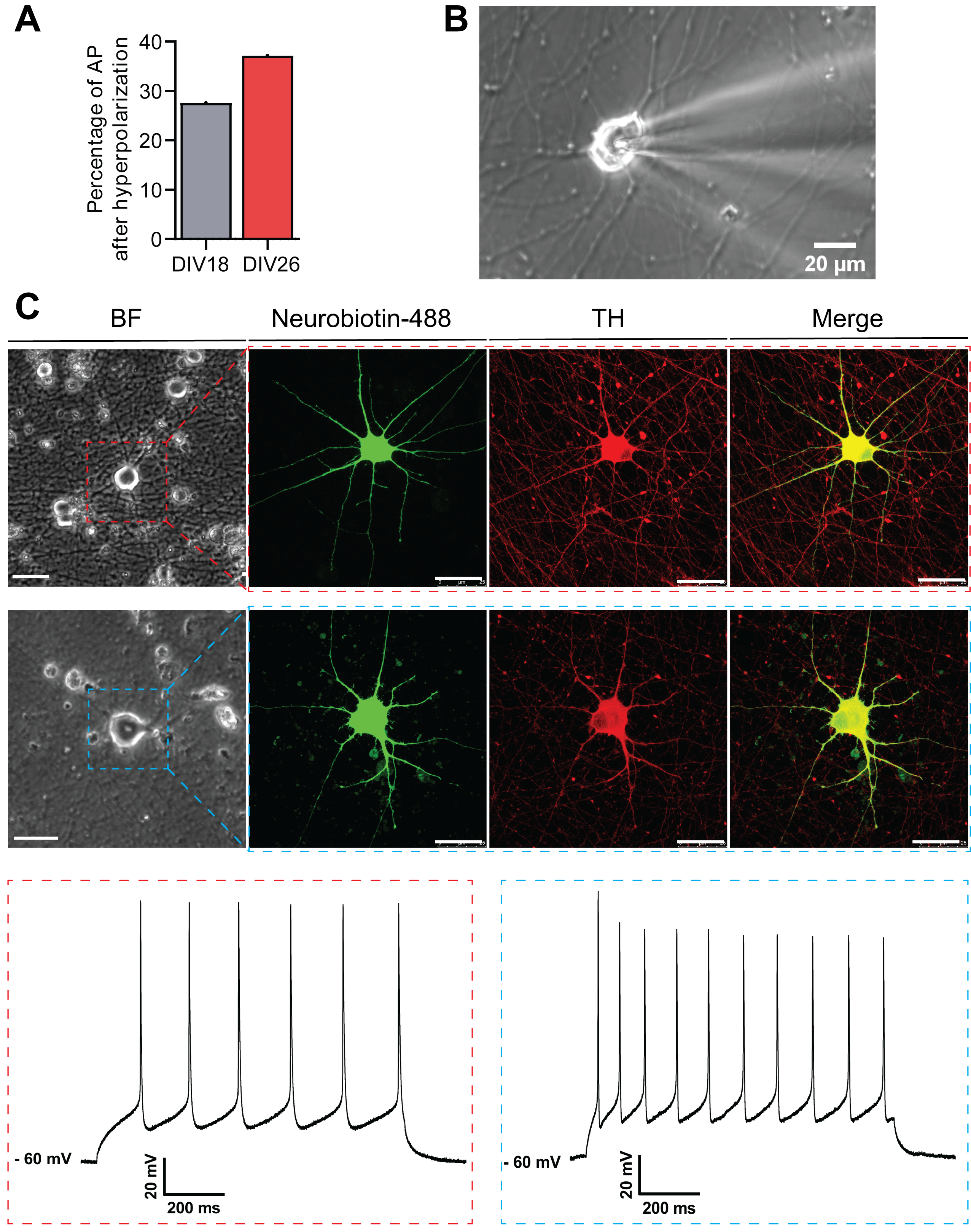

Supplement: Supplementary file 6 — Supplementary Information 6. [file 41598_2022_22158_MOESM6_ESM.tif]
