# Supplementary material for: Combining NGN2 programming and dopaminergic patterning for a rapid and efficient generation of hiPSC-derived midbrain neurons
Source: Sci Rep. 2022 Oct 13;12:17176. doi: 10.1038/s41598-022-22158-4 (PMC9562300; doi:10.1038/s41598-022-22158-4)
Supplement: Supplementary file 7 — Supplementary Information 7. [file 41598_2022_22158_MOESM7_ESM.pdf]

Figure S4C-raw images

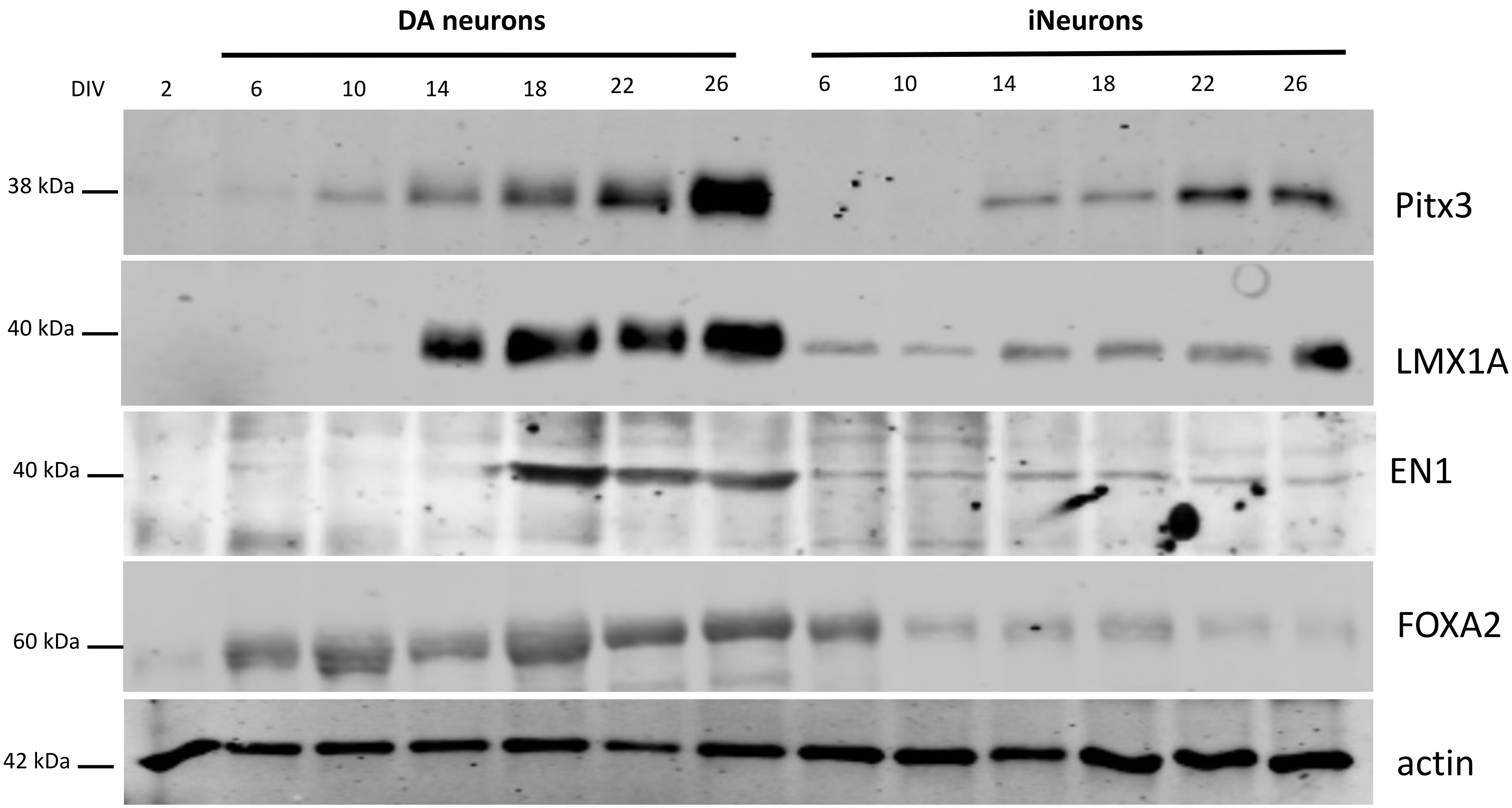
DA neurons
iNeurons
DIV
2
6
10
14
18
22
26
6
10
14
18
22
26
38 kDa
40 kDa
40 kDa
60 kDa
42 kDa
Pitx3
LMX1A
EN1
FOXA2
actin

Figure S1-A_PITX3

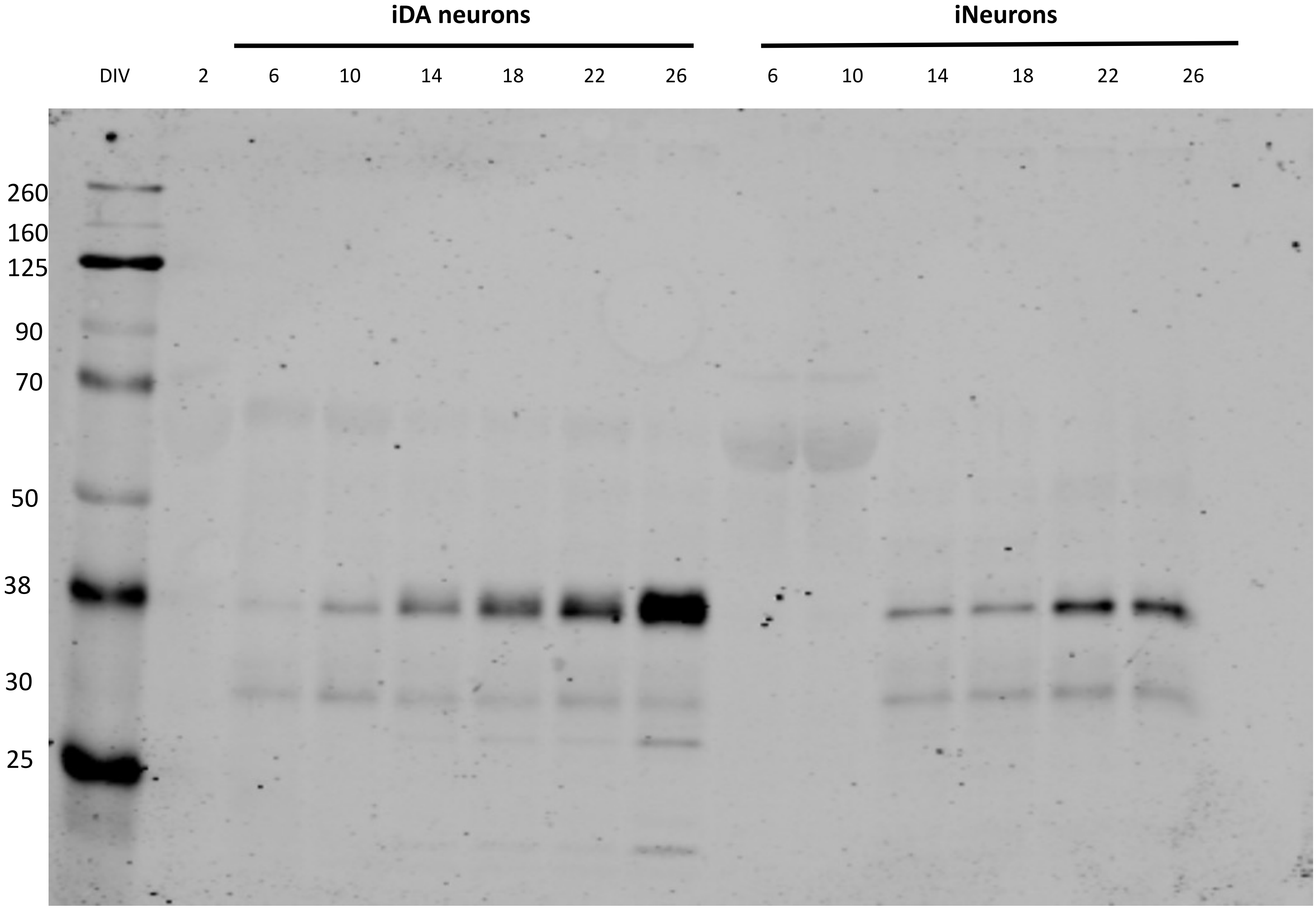

Visualization was carried out with the LI-COR Odyssey scanner and software (LI-COR Lincoln, NE, USA).

Figure S1-A_LMX1

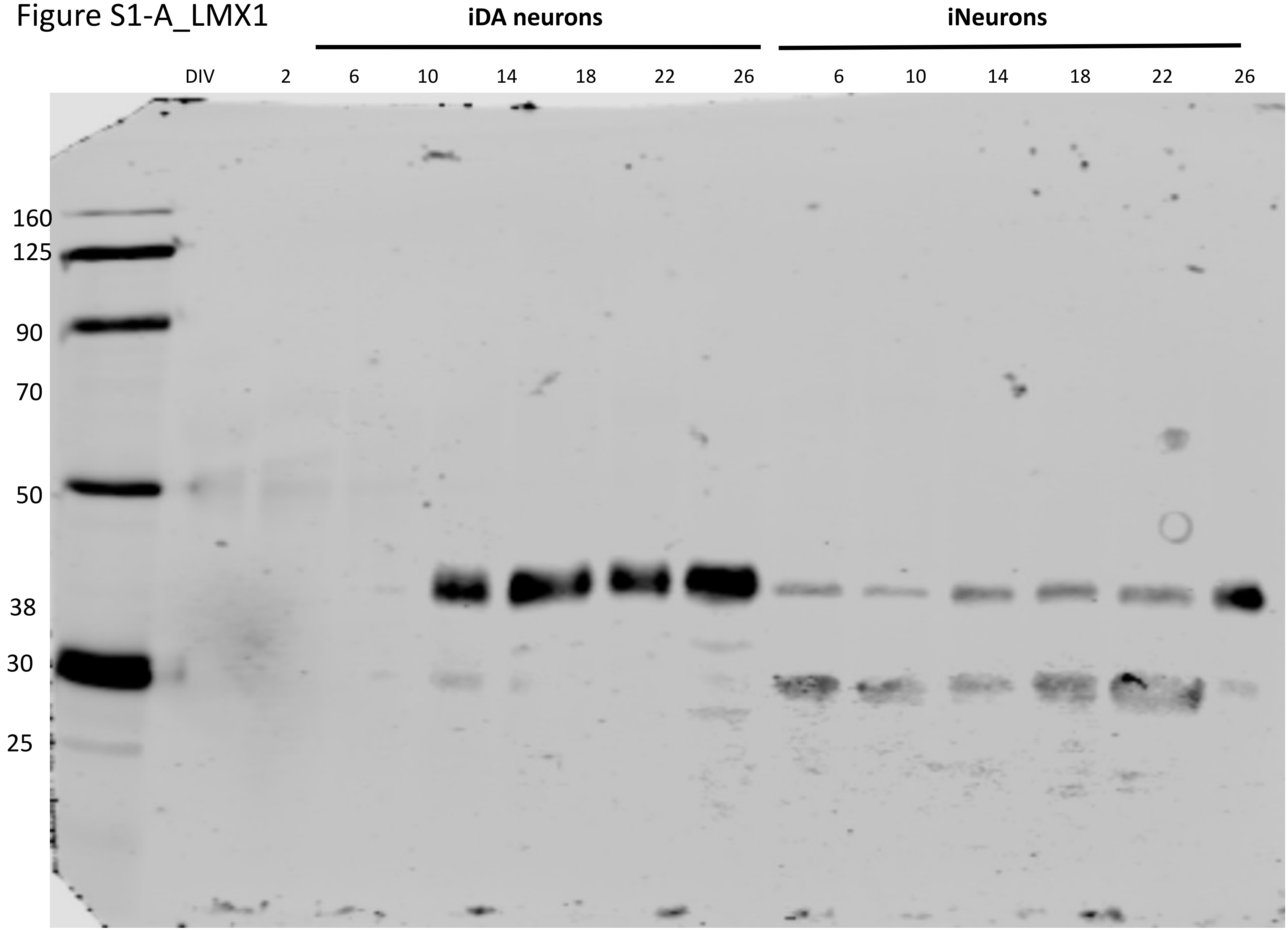

Visualization was carried out with the LI-COR Odyssey scanner and software (LI-COR Lincoln, NE, USA).

Figure S1-A_EN1

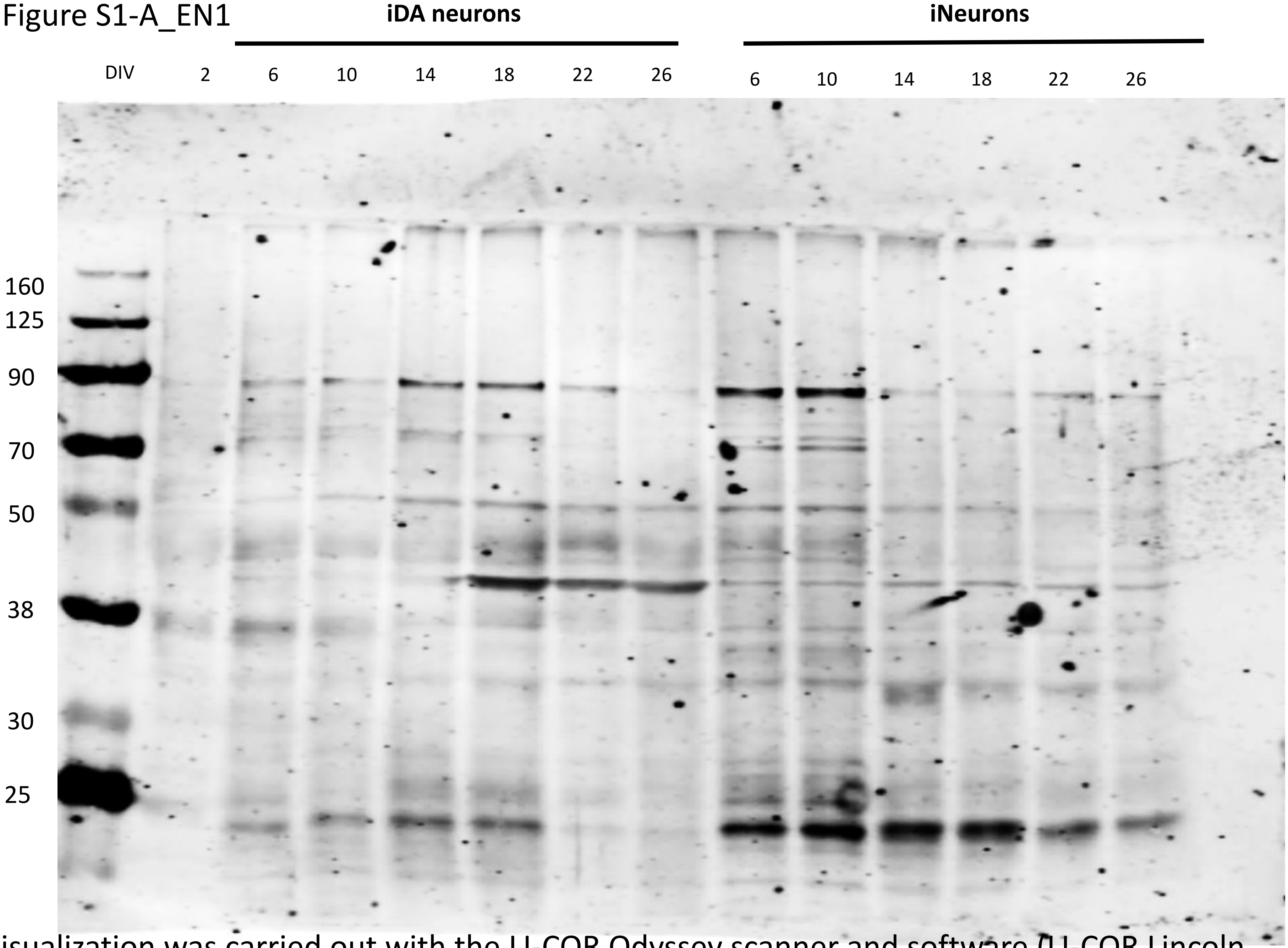

Visualization was carried out with the LI-COR Odyssey scanner and software (LI-COR Lincoln, NE, USA).

Figure S1-A_FOXA2

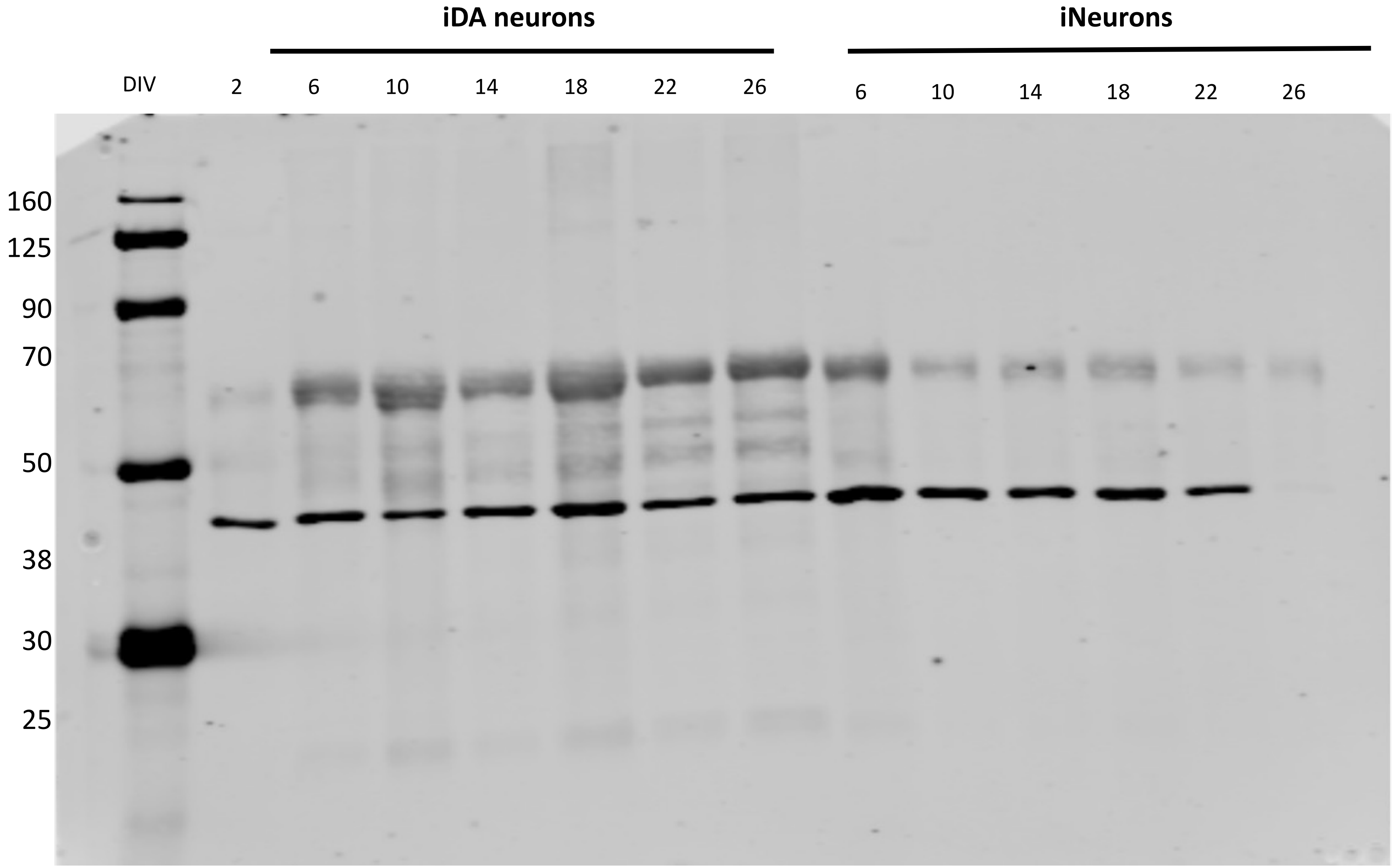

Visualization was carried out with the LI-COR Odyssey scanner and software (LI-COR Lincoln, NE, USA).

Figure S1-A_actin

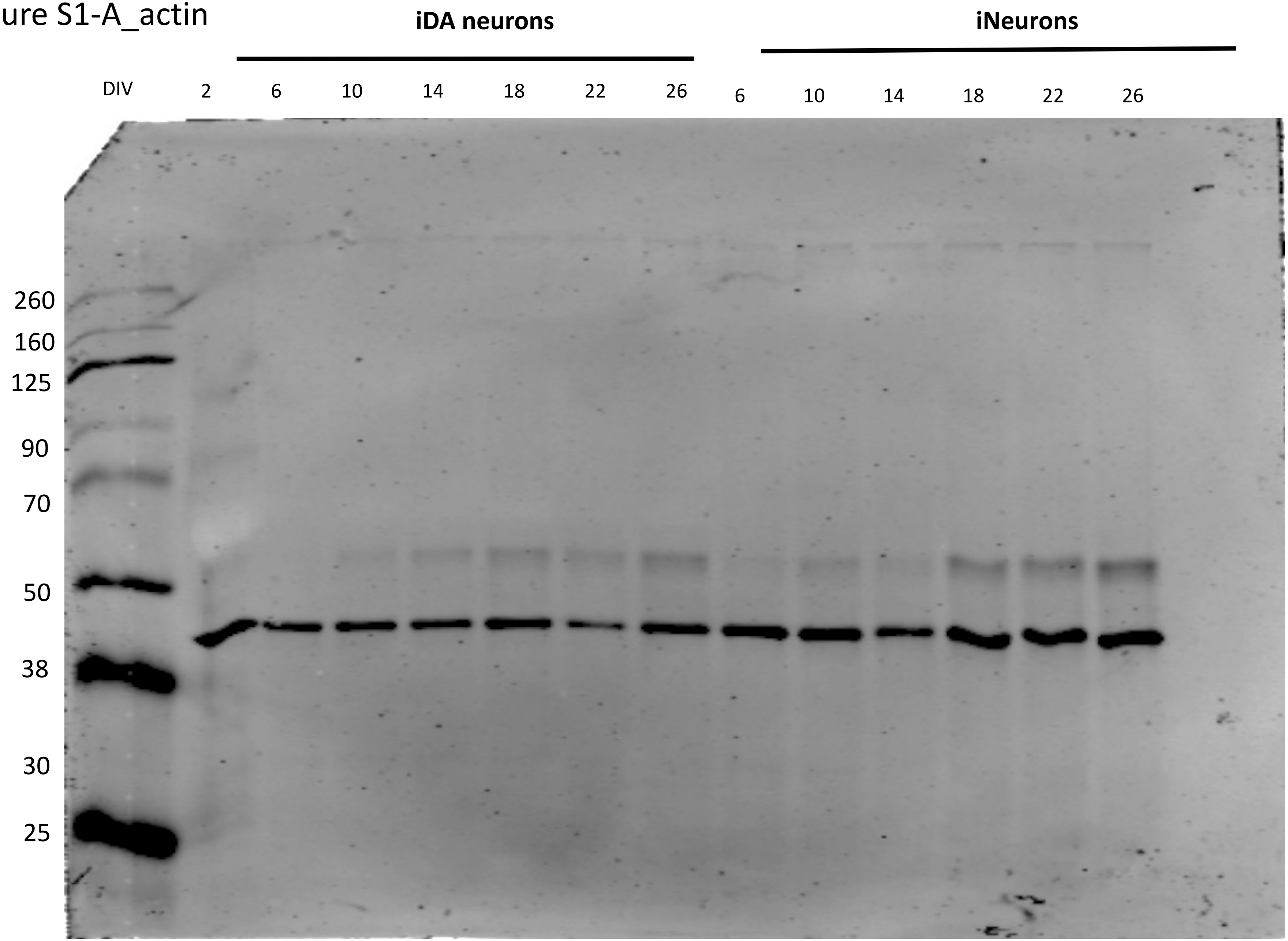

Visualization was carried out with the LI-COR Odyssey scanner and software (LI-COR Lincoln, NE, USA).
